# Transoceanic pathogen transfer in the age of sail and steam

**DOI:** 10.1073/pnas.2400425121

**Published:** 2024-07-16

**Authors:** Elizabeth N. Blackmore, James O. Lloyd-Smith

**Affiliations:** ^a^Department of Ecology and Evolutionary Biology, University of California, Los Angeles, CA 90095; ^b^Department of Ecology and Evolutionary Biology, Yale University, New Haven, CT 06520

**Keywords:** theoretical modeling, global disease history, transmission dynamics, pathogen ecology

## Abstract

In the centuries following Christopher Columbus’s 1492 voyage to the Americas, transoceanic travel opened unprecedented pathways in global pathogen circulation. Yet no biological transfer is a single, discrete event. We use mathematical modeling to quantify historical risk of shipborne pathogen introduction, exploring the respective contributions of journey time, ship size, population susceptibility, transmission intensity, density dependence, and pathogen biology. We contextualize our results using port arrivals data from San Francisco, 1850 to 1852, and from a selection of historically significant voyages, 1492 to 1918. We offer numerical estimates of introduction risk across historically realistic ranges of journey time and ship population size, and show that both steam travel and shipping regimes that involved frequent, large-scale movement of people substantially increased risk of transoceanic pathogen circulation.

## Significance

Five hundred years ago, Christopher Columbus’s voyage to the Americas opened a new era of global pathogen exchange. Yet the globalization of infectious disease that followed was neither rapid nor universal. We use mathematical modeling to investigate how easily pre-20th century ships carried pathogens across oceans. We show that shipping practices that involved frequent, large-scale people-movement, and novel technologies such as steam travel both increased the likelihood of transoceanic pathogen transfer. These results challenge longstanding narratives of rapid and inevitable pathogen unification across oceans. They offer a rigorous, biologically informed framework for investigating the broader contours of global pathogen history. Finally, our study offers a timely reminder of the emergence of global pathogen ecosystems centuries before present-day air travel.

In the centuries following Christopher Columbus’s 1492 journey to the Americas, transoceanic voyages opened unprecedented pathways in global pathogen circulation. In 1962, historian Woodrow Borah described the changes that followed as near-immediate; in his account, previously isolated regions such as the Americas and the Pacific “received within a few decades the united impact of all the diseases that could be spread” (1). This narrative of rapid—and inevitable—pathogen transfer continues to shape some popular histories of global infectious disease (2). Yet while transoceanic shipping was indeed a pivotal ecological force, the changes that followed took substantially longer than decades. Global pathogen transfer was—and is—a centuries-long process, starting in 1492 but subsequently transformed by mass migration, by the 19th-century steam revolution, and—most recently—by contemporary air travel.

Sixty years on from Borah’s interpretation, scholars in both the humanities and the sciences have charted the slow globalization of infectious disease. In the 1970s, pioneering environmental histories such as Alfred Crosby’s “Columbian Exchange,” Emmanuel Le Roy Ladurie’s “microbial unification of the world,” and William McNeill’s “common market of microbes” expanded the scope of Borah’s analyses to show that first introductions of “Old World” pathogens into previously isolated regions spanned one or two centuries following European arrival (3–5). Subsequent historians have shown that pathogen exchange across the Atlantic, Pacific, and Indian oceans occurred slowly, with some introductions causing only transient outbreaks (2, 6–13). These outcomes were highly contingent on local human processes such as trade, warfare, and colonialism. In parallel, disease ecologists have shown that acute pathogens such as measles and influenza require large human populations for endemic local establishment (14–17), and that in smaller populations, continued circulation depends on regular introductions from “source” populations (18, 19). These metapopulation dynamics were as critical to historical pathogen dynamics as they are today (20–25). Throughout the 18th century, the city of Boston, Massachusetts experienced decades-long intervals between smallpox outbreaks (26, 27), while the much larger city of London experienced smallpox cases every year since records began in 1664 (27). Sporadic introductions were, and are, extremely impactful—particularly in populations with no prior immunity (28–30). Sustained “microbial unification”—the transition from intermittent introduction to continuous global circulation—required regular human movement and, with it, continued introduction and reintroduction of pathogens.

This raises an ecological question. How easily did infectious diseases survive the weeks- or months-long voyages necessary for transoceanic pathogen transfer in the age of sail and steam? There is good reason to expect that transoceanic pathogen introduction under these conditions was far from assured—particularly for fast-burning respiratory infections such as smallpox, measles, and influenza. As late as the 1850s, a sail voyage from Liverpool to New York City could take 5 to 6 wk (31), while journeys from the UK to Australia could take 3 to 4 mo (32). Between lengthy periods at sea, short infection generation times, and intense shipboard transmission, fast-burning “crowd diseases” could rapidly exhaust all susceptible people on board and go extinct long before a vessel reached port, leaving no pathogen to introduce.

We explore the mechanics of shipborne pathogen transfer using the toolkit of contemporary theoretical ecology. We present a stochastic Susceptible-Exposed-Infectious-Recovered (SEIR) model which quantifies the probability of an outbreak lasting a given duration in a closed population. We consider the relative contributions of a broad range of factors to outbreak duration, including pathogen natural history, transmission intensity and density dependence, population size, and population susceptibility. Finally, we use port arrivals data from Gold Rush-era San Francisco, California, 1850 to 1852, to explore the implications of variation in journey length, ship size, and natural history for pathogen circulation in the specific context of the Pacific. As part of this, we explore the impact of the advent of steam travel in the 19th century—a technological revolution that routinely cut journey times by a factor of two or more (31–33).

The idea that many shipboard outbreaks ended long before a vessel’s arrival is intuitive. These processes have been considered qualitatively, both by scientists (33, 34) and by historians (3, 10, 13, 35). Paterson et al. (36) have also modeled the specific case of shipborne measles introductions to Australia during the 19th century. A more general quantitative analysis can offer sharper insight into the contours of global disease history, and can aid in building broader structural histories of infectious disease (9, 13, 37, 38). It can also reveal new patterns in seemingly disparate disease introductions.

Our results indicate that shipborne pathogen introductions were neither trivial nor inevitable. Ships were not simple pathogen vectors: They were populations. The extinction and survival dynamics of pathogens on ships were complex population biological processes, contingent on pathogen natural history and host population size, composition, and mixing patterns. Thus, the history of transoceanic disease introduction is a story both of fundamental pathogen biology, and of human economics, technology, and behavior. Theoretical modeling can reveal how these forces interacted to shape global disease transmission.

## Results

### Basic Dynamics.

**Fig. 1.**
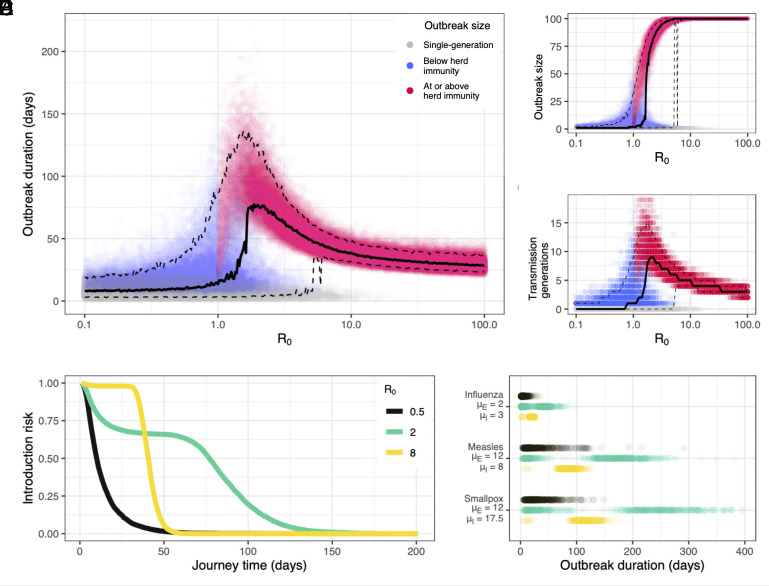
Basic dynamics. (*A*) Outbreak duration, (*B*) outbreak size and (*C*) number of transmission generations by $R_{0}$, assuming a fully susceptible population of N=100 and a theoretical pathogen with $\mu_{E}=\mu_{I}=\;5\;\text{d}$ and $k_{E}=k_{I}=3$. Solid black lines show median outbreak duration, outbreak size, and number of generations. Top and bottom dashed lines respectively show 95th and fifth percentile outbreak duration, outbreak size, and number of generations. (*D*) Probability of at least one person in state E or state I (“introduction risk”) for any given journey time by $R_{0}$, using the same population and pathogen parameters. (*E*) Outbreak length distribution by $R_{0}$ in a fully susceptible population of N=100 for influenza, measles, and smallpox, using epidemiological parameters detailed in *SI Appendix*, Table S1.

Transoceanic pathogen introduction requires a chain of infections that lasts at least as long as a ship’s journey time. To investigate the basic dynamics of shipboard outbreak duration, we simulate outbreaks in a fully susceptible population (N=100) using a hypothetical pathogen which has characteristics typical of acute respiratory viruses (mean incubation and infectiousness periods of 5 d each) (Fig. 1). We define outbreak duration as the time until nobody on board ship is infected with the pathogen, that is, until E=I=0.

Historical accounts indicate that transmission on board ships was substantially more intense than transmission in typical land settings (*SI Appendix*, *Text S1*). Thus, we explore a broad range of transmission intensities. These are summarized by the epidemiological parameter $R_{0}$, or the average number of infections that a single person will produce in a fully susceptible population.

We observe three outbreak duration regimes, all of which depend heavily on transmission intensity. Under strongly subcritical transmission ($R_{0}≲0.8$), the majority of simulations result in no transmission beyond the index case (Fig. 1*A*). These “single-generation” outbreaks last only as long as the course of infection in a single person, in this case, an average of 10 d.

Under strongly supercritical transmission ($R_{0}≳5$), outbreaks are large and almost universally reach or exceed the threshold for ship herd immunity, $\frac{S}{N}<\frac{1}{R_{0}}$ (Fig. 1*B*). This reliably results in 35 to 55 d outbreaks for the modeled scenario, with duration steadily decreasing as $R_{0}$ increases. Occasionally, simulations under strongly supercritical $R_{0}$ also result in short single- or two-generation outbreaks (Fig. 1 *A* and *C*); these are consistent with the occurrence of minor outbreaks due to random extinction in stochastic systems (39, 40).

Values of $R_{0}$ near criticality ($0.8≲R_{0}≲5$), produce the longest outbreaks, with median duration peaking around $R_{0}=2$. These are made possible by extended, multigenerational transmission chains (Fig. 1*C*). Yet while near-critical conditions give rise to the longest outbreaks, they also result in the widest range of outbreak durations. An $R_{0}$ of 1 may result in outbreaks that last 150 d or more, but median outbreak duration under these conditions is just 14 d.

Transmission intensity modulates a pathogen’s overall introduction risk—here defined as the net probability that at least one passenger is carrying the pathogen (i.e. in state E or state I) upon arrival, for any given journey length. Under strongly subcritical transmission (e.g. $R_{0}=0.5$), introduction risk decays rapidly with journey time, with 50% probability of introduction at 10 d and 25% probability at 17 d (Fig. 1*D*). Under strongly supercritical transmission ($R_{0}=8$), pathogen introduction risk is sigmoidal: Introduction is near certain (≥95%) for journeys of 33 d or less, then falls rapidly for journey times exceeding this threshold. Introduction is least predictable for weakly supercritical values of $R_{0}$ ($R_{0}=2$). Here, many outbreaks end quickly due to random extinction (Fig. 1*A*). Yet past this threshold, risk broadly plateaus until much longer journey times (∼60 d), then declines with a long tail. Thus, the relative introduction risk of weakly versus strongly supercritical transmission depends on journey length. Strongly supercritical transmission is significantly more likely to result in pathogen introduction for journeys of 33 d or less, since intense transmission carries minimal risk of early extinction. But across journeys of 40 d or more, pathogen introduction is most likely under weakly supercritical transmission.

For real pathogens, introduction thresholds are governed by pathogen-specific natural history, above all by the durations of a pathogen’s latent and infectious periods (Fig. 1*E*). We explore outbreak length for influenza, measles, and smallpox at subcritical, near-critical, and strongly supercritical $R_{0}$ (*SI Appendix*, Table S1). The results demonstrate that relative introduction risk can be often inferred from pathogen natural history, even in the absence of shipboard $R_{0}$ estimates. At any $R_{0}$, smallpox typically survives longer on board a ship than measles, which in turn typically survives longer than influenza. Natural history also indicates some general introduction thresholds, which hold regardless of transmission intensity. For example, for a ship with 100 people on board, influenza introduction is extremely unlikely for journeys lasting longer than 100 d, regardless of $R_{0}$.

### Incorporating Population Size and Susceptibility.

Next, we expand our analysis beyond the unlikely scenario of one ship with N=100 and 100% population-level susceptibility to consider the combined effects of ship population size, N, and initial proportion susceptible, $\frac{S(0)}{N}$, on ship outbreak duration.

In populations with some initial immunity to infection (i.e. where $\frac{S(0)}{N}<1$), transmission intensity is most meaningfully measured as a pathogen’s “effective” reproduction number, $R_{e}$. Because population immunity levels change over the course of an outbreak, this is commonly expressed as a function of time, i.e. $R_{e}\left(t\right)$. Notably, $R_{e}\left(t\right)$ is a linear function of a pathogen’s basic reproduction number, $R_{0}$. Hence, $R_{e}\left(t\right)=\frac{S(t)}{N}R_{0}$, with critical transmission occurring at the threshold $R_{e}\left(t\right)=1$. We consider shipboard transmission at t=0, where $R_{e}\left(0\right)=\frac{S(0)}{N}R_{0}$.

**Fig. 2.**
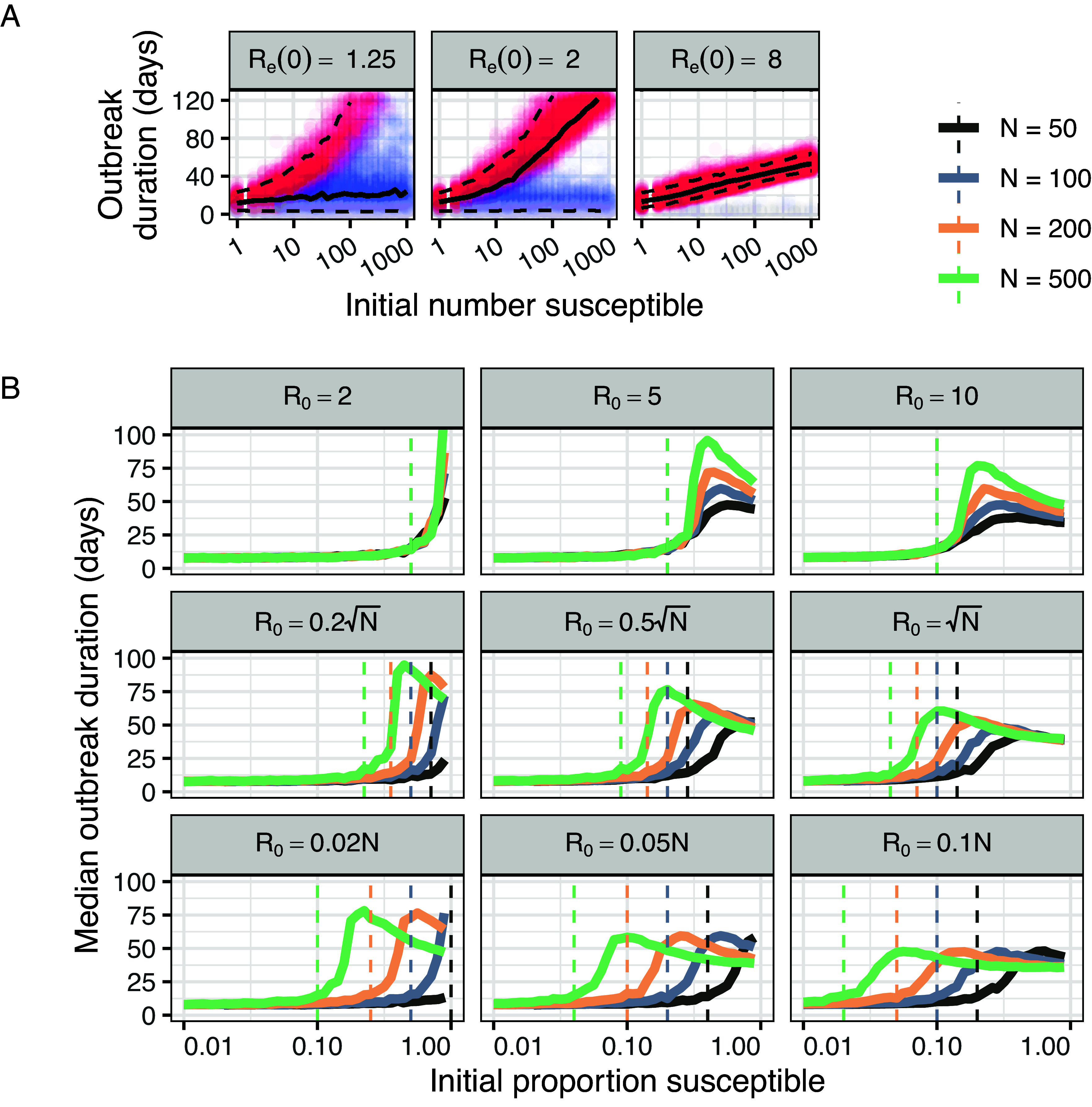
Effect of susceptible population size. (*A*) Outbreak duration by initial susceptible population (S(0)) and effective reproduction number ($R_{e}\left(0\right)$). We fix N= 1,001 and backcalculate $R_{0}$ for each value of S(0) to maintain constant $R_{e}\left(0\right)$. As above, we base simulations on a theoretical pathogen with $\mu_{E}=\mu_{I}=5$ d. Solid black lines show median outbreak duration; top and bottom dashed lines show 95th and fifth percentile durations respectively. Colors match those used in Fig. 1*A*–*C* and denote single-generation outbreaks (gray), outbreaks that reach herd immunity (red), and outbreaks that terminate before herd immunity is achieved (blue). (*B*) Median outbreak duration by ship size and by initial proportion susceptible. Rows show three density dependence scenarios: full frequency dependence (q=0, Top *row*); intermediate density dependence (q=0.5, Middle *row*); and full density dependence (q=1, Bottom *row*). Columns show three scenarios for transmission intensity: $\beta_{\mathrm{fd}}=0.04$ (*Left*); $\beta_{\mathrm{fd}}=1$ (*Middle*); and $\beta_{\mathrm{fd}}=2$ (*Right*). Calculated using $\mu_{E}=\mu_{I}=5$ d and $\beta_{\mathrm{dd}}=\beta_{\mathrm{fd}}/100$ (*Materials and Methods*; *SI Appendix*, *Text S1D*).

First, we vary the total number of people who are initially susceptible, S(0), while holding $R_{e}\left(0\right)$ constant. We do so by fixing N= 1,001, choosing S(0), and backcalculating $R_{0}$ to maintain the same effective rate of transmission. This results in a roughly log–linear relationship between initial susceptible population size and outbreak duration at near-critical and supercritical values of $R_{e}\left(0\right)$ (Fig. 2*A*). At $R_{e}\left(0\right)=1.25$, increasing S(0) has little influence on median outbreak duration but substantially increases 95th percentile outbreak duration. At $R_{e}\left(0\right)=2$, increasing S(0) increases both median and 95th percentile outbreak times. Finally, at $R_{e}\left(0\right)=8$, increasing S(0) dependably increases median, fifth percentile, and 95th percentile outbreak times.

Next, we vary N as well as S(0). This opens the question of what relationship we expect between N, S, and $R_{0}$ in the unique environment on board a historical ship. Records from the time indicate that many vessels suffered from inadequate ventilation and extreme levels of crowding (*SI Appendix*, *Text S1*). On land, these conditions generally give rise to “density-dependent” patterns of transmission, where contact rates scale linearly with population size ($R_{0}\propto N$). Yet ships were also famously structured and compartmentalized environments, which typically align with assumptions of “frequency-dependent” transmission (*SI Appendix*, *Text S1*); here contact rates are assumed to remain constant, regardless of total population size ($R_{0}╨\;N$).

In practice, we expect that effective density dependence varied substantially according to ship layout and construction, social norms, and pathogen-side biology. Thus we consider three density dependence scenarios: classical density dependence ($R_{0}\propto N$), classical frequency dependence ($R_{0}╨\;N$), and an intermediate degree of density dependence ($R_{0}\propto N^{0.5}$). Under each scenario, we explore the effect of initial ship susceptibility, $\frac{S(0)}{N}$, on median outbreak duration across several total population sizes, N.

In all circumstances, larger and more susceptible populations generally present greater risks of pathogen introduction across any given journey (Fig. 2*B*). Yet they do so in different ways, and for different reasons.

Under classical frequency dependence, $R_{0}=\mu_{I}\beta_{\mathrm{fd}}$, where $\mu_{I}$ represents the average duration of an individual’s infectious period and where $\beta_{\mathrm{fd}}$ represents the average number of onward infections that a single infected person would generate, per day, in a fully susceptible population. Critical transmission, $R_{e}\left(0\right)=1$, occurs at the constant threshold $\frac{S(0)}{N}={\left(\mu_{I}\beta_{\mathrm{fd}}\right)}^{-1}$. The threshold value of $\frac{S(0)}{N}$ required for $R_{e}\left(0\right)=1$ is independent of total ship population, N. However, for any given $\frac{S(0)}{N}$, ships with greater N must have a proportionally greater number of susceptibles, S(0). Since ships with greater S(0) experience longer outbreaks at a given supercritical value of $R_{e}\left(0\right)$ (Fig. 2*A*), ships with greater total populations display longer median outbreak times at $R_{e}\left(0\right)>1$ (Fig. 2*B*, *Top* *row*).

Under classical density dependence, $R_{0}=\mu_{I}\beta_{\mathrm{dd}}N$, where $\beta_{\mathrm{dd}}$ represents the average proportion of a given population, N, that a single infected person would infect per day in a fully susceptible population. Critical transmission occurs at the threshold $\frac{S(0)}{N}={\left(\mu_{I}\beta_{\mathrm{dd}}N\right)}^{-1}$. Multiplying both sides by a factor of N reveals that critical transmission depends solely on initial susceptible population size: $S\left(0\right)={\left(\mu_{I}\beta_{\mathrm{dd}}\right)}^{-1}$. When N is large, this threshold for S(0) represents a smaller fraction of the total population. But, in contrast to frequency-dependent transmission, S(0) is constant at any given $R_{e}\left(0\right)$, and so peak outbreak duration does not vary across ships of different sizes. Rather, larger ship populations give rise to near-critical and supercritical transmission at lower values of $\frac{S(0)}{N}$ (Fig. 2*B*, *Bottom* *row*).

Under intermediate transmission, $R_{0}=\mu_{I}{\left(\beta_{\mathrm{fd}}\beta_{\mathrm{dd}}N\right)}^{0.5}$. Critical transmission occurs at the threshold $\frac{S(0)}{N}=\mu_{I}^{-1}{\left(\beta_{\mathrm{fd}}\beta_{\mathrm{dd}}N\right)}^{-0.5}$, and at the total susceptibility level $S\left(0\right)=N^{0.5}\mu_{I}^{-1}{\left(\beta_{\mathrm{fd}}\beta_{\mathrm{dd}}\right)}^{-0.5}$. Under this model, larger ship populations reach critical transmission at slightly lower initial proportions of susceptibility, have a critical S(0) that scales sublinearly with N, and hence display slightly higher outbreak length for any given $R_{e}\left(0\right)$ (Fig. 2*B*, *Middlerow*).

Finally, we note that regardless of density dependence, ships with a higher rate of contact (represented either by $\beta_{\mathrm{fd}}$ or by $\beta_{\mathrm{dd}}$) require lower initial susceptibility for critical transmission (compare across rows in Fig. 2*B*). Thus, ships with higher rates of social mixing (e.g. more crowded ships) require fewer susceptible people to achieve supercritical transmission, regardless of total ship population size.

Thus, even in the absence of detailed reconstructions of ship transmission patterns, we can conclude that ships with larger, more crowded populations presented greater risks of pathogen introduction—be this by increasing total persistence times, decreasing the susceptibility fraction required for critical transmission, or both. In practice, the risk associated with larger ship populations was almost certainly boosted further by an increased chance of carrying at least one infected person on departure. We do not account for this difference, instead conditioning our analysis on the assumption that each ship departs with a single infected individual. Yet in cases with low infection prevalence at the port of origin, this elevated chance of having at least one infected person on board at the time of departure would have substantially increased net introduction risk. Thus, ships with larger populations were both more likely to depart with infection on board and more likely to sustain this infection outbreak until arrival.

### Historical Applications.

**Fig. 3.**
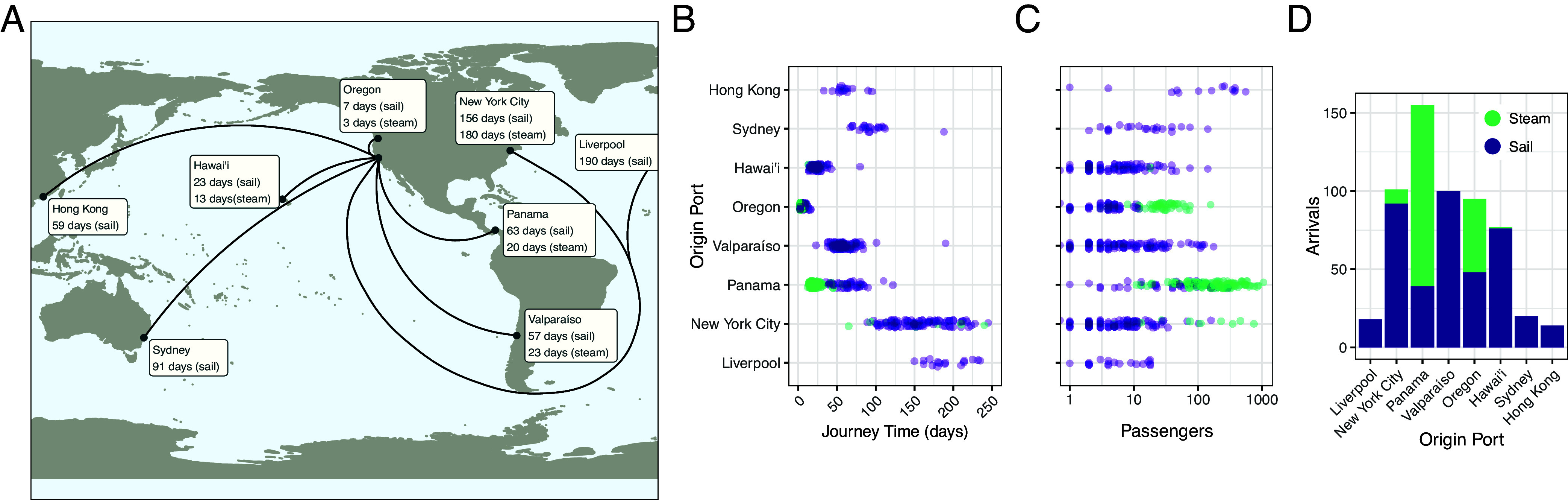
San Francisco arrivals, June 1850 to June 1852. (*A*) Map of arrivals into San Francisco harbor, June 6, 1850 to June 9, 1852, with median journey times by ship technology. (*B*) Journey time, (*C*) passenger number and (*D*) number of voyages by origin port and by ship technology. Data from Louis J. Rasmussen’s *San Francisco Passenger Lists* (41–43).

Voyage characteristics such as journey time, population size, and population susceptibility varied substantially across different time periods, transit routes, and ship constructions. We explore some of this variation, and its implications, in the context of the Pacific basin. Specifically, we use port arrivals data for Gold Rush-era San Francisco, 1850 to 1852, originally collected by historian and genealogist Louis J. Rasmussen (Fig. 3) (41–43).

While acute respiratory infections first crossed the Atlantic Ocean in the late 15th and early 16th centuries (13), by the mid-19th-century pathogens such as smallpox and measles had only recently begun to arrive across the Pacific basin. California saw its first region-wide outbreaks of measles and smallpox in 1806 and 1838, respectively (44–46). Smallpox was first introduced to Australia in 1788 but did not see second introduction until 1829, while measles appears to have arrived for the first time in 1850 (36, 47). Several Pacific islands saw initial introductions well into the late 19th century, including Hawai’i (smallpox, 1853); Easter Island (smallpox, 1863); Fiji (measles, 1875); and Tonga (measles, 1893) (47).

During the years 1850 to 1852, passengers journeyed to San Francisco from across East Asia, Australasia, South America, and Europe. In an era preceding reliable transcontinental rail, ocean travel also provided one of the fastest and safest routes from eastern North America to the newly established state of California (48). Median sailing times ranged from 7 d (from Oregon Territory) to 190 d (from Liverpool, England), with considerable variation within routes (Fig. 3 *A* and *B* and *SI Appendix*, Table S2). Longer-range sail voyages displayed an especially broad range of transit times. For example, sail journeys from New York City could be as long as 283 d (on the *Primoguet*) or as short as 89 d (on the *Flying Cloud* —reportedly “the fastest [sail] voyage ever recorded”) (42).

Steam travel represented a phase transition in transoceanic pathogen circulation for several reasons. First, in most cases, the technology dramatically reduced journey times. Median transit times from Panama were 63 d by sail but just 20 d by steam. Meanwhile, steam reduced median journey times from Oregon from 7 d to just 3 d (*SI Appendix*, Table S2). These shorter journey times would have increased risk of shipborne pathogen introduction significantly.

Second, steam ships transported some of the greatest numbers of passengers (Fig. 3*C*). Steamers from Panama carried a median of 196 passengers and as many as 1,050, compared with a median of 53 and a maximum of 287 by sail (*SI Appendix*, Table S2). Oregon steamers carried a median of 28 passengers and as many as 157, compared with a median of 4 and a maximum of just 12 by sail. Finally, steam vessels from New York City carried a median of 111 passengers and a maximum of 743, compared to a median of 5 and a maximum of 160 by sail. The only sail route that could compete with steam travel on passenger numbers was the route from Hong Kong, which transported a median of 201 passengers and a maximum of 553. As demonstrated above, larger passenger numbers would have substantially increased ships’ capacities for sustained pathogen circulation.

Finally, steam travel represented some of the most frequent voyages (Fig. 3*D*), resulting in a greater cumulative risk of pathogen introduction across any given period. Particularly striking are the 116 steam journeys from Panama that arrived between June 1850 and June 1852.

**Fig. 4.**
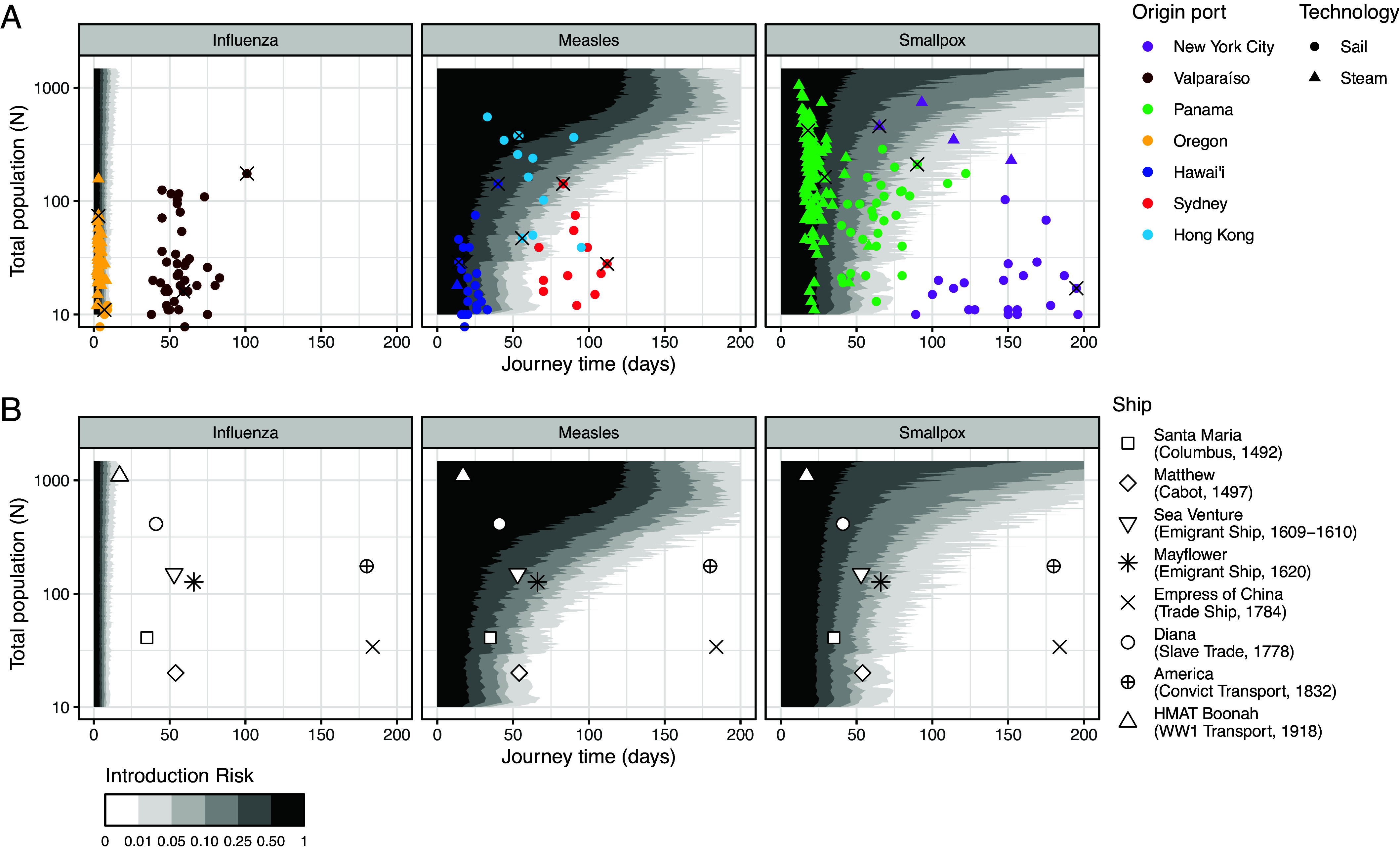
Historical applications. Introduction risk for influenza, measles, and smallpox by journey time and by total ship population, N, assuming 5% initial population-level susceptibility, intermediate density dependence (q=0.5), and $\mu_{E}$, $\mu_{I}$, and $\beta_{\mathrm{fd}}$ according to consensus natural history parameters (*SI Appendix*, Table S1). We backcalculate $\beta_{\mathrm{fd}}$ as $1/\mu_{I}$ times a pathogen’s typical land $R_{0}$, and set $\beta_{\mathrm{dd}}=\beta_{\mathrm{fd}}/100$ (*SI Appendix*, *Text S1D*). (*A*) overplots data on San Francisco Port arrivals, June 1850 to June 1852. Here, total population (N) represents only the passengers on board each ship, as crew data are not available. Introduction risks for all three pathogens are shown for 15 selected voyages in Table 1; these voyages are indicated with black crosses. For the two ships with documented infectious disease outbreaks, the *Gold Hunter* and the *Sir Charles Napier*, we also performed sensitivity analyses investigating the robustness of our predictions to different rates of transmission and population-level susceptibility (*SI Appendix*, Fig. S1). (*B*) overplots selected historical journeys, 1492 to 1918, chosen to be indicative of the broad trends in transoceanic shipping. N represents the combined totals of passengers and crew. Sources and further data are available in *SI Appendix*, Table S3. Numerical introduction risk estimates for (*B*) are provided in Table 2. HMAT Boonah (WW1 Transport, 1918) refers to His Majesty’s Australian Transport Boonah, a troop ship used in the First World War.

To explore possible differences in introduction risk across each route and type of ship, we simulate influenza, measles, and smallpox outbreaks across the full range of ship populations and journey times represented in the San Francisco dataset (Fig. 4*A*). Here, contours represent pathogen introduction risk by journey time and by total ship population, assuming 5% population-level susceptibility and intermediate density dependence, and calibrating transmission intensity with reference to standard literature values and analyses of shipboard outbreaks (*SI Appendix*, *Text S1D* and Table S1). We overplot individual journeys into San Francisco to assess pathogen introduction risk across each route (Fig. 4*A*). We plot a selection of routes on each panel for visual clarity. However, the observations below concern introduction risk for all pathogens across all routes traveled.

For select voyages, we also provide numerical introduction risk estimates for each pathogen in Table 1. Particularly interesting are two voyages from Panama with documented outbreaks of acute viral infections: the *Gold Hunter* steam ship, which arrived in San Francisco after a 29-d voyage with one active case of smallpox, and the *Sir Charles Napier* sail ship, which experienced an outbreak of “measles, dysentery, and fever” lasting “about 3 wk’ of its 90-d voyage.

**Table 1.** Numerical introduction risk estimates for influenza, measles, and smallpox across selected voyages to San Francisco, 1850 to 1852

				Pathogen introduction risk		
From	Type	Days	N	Influenza	Measles	Smallpox
New York	Sail	195	17	<0.001	<0.001	<0.001
New York	Steam	65	458	<0.001	0.611	0.207
Valparaíso	Sail	59	16	<0.001	0.010	0.036
Valparaíso	Sail	101	175	<0.001	0.053	0.013
Panama*	Sail	90	211	<0.001	0.160	0.057
Panama†	Steam	29	163	<0.001	0.556	0.422
Panama	Steam	18	420	0.001	0.813	0.749
Oregon	Steam	3	74	0.656	0.996	>0.999
Oregon	Sail	7	11	0.095	0.931	0.979
Hawai’i	Sail	40	142	<0.001	0.399	0.254
Hawai’i	Sail	14	29	0.001	0.692	0.817
Sydney	Sail	112	28	<0.001	<0.001	<0.001
Sydney	Sail	83	142	<0.001	0.076	0.032
Hong Kong	Sail	56	47	<0.001	0.040	0.047
Hong Kong	Sail	54	377	<0.001	0.613	0.224

^*^This vessel, the *Sir Charles Napier*, experienced an outbreak of “measles, dysentery, and fever” which lasted “about 3 wk.” 36 passengers died, with the final death occurring 54 d into the voyage (53) (*SI Appendix*, Table S4 and Fig. S1).

^†^This vessel, the *Gold Hunter*, arrived in San Francisco with one active smallpox case. The patient was isolated on arrival (54) (*SI Appendix*, Table S4 and Fig. S1).

Influenza has very short latent and infectious periods ($\mu_{E}=2$d, $\mu_{I}=3$d) and a relatively low $R_{0}$ (around 1.5) (49–51). Together, these result in a very low risk of introduction into San Francisco from any origin port except Oregon and perhaps Panama and Hawai’i. Even then, only the fastest voyages presented any significant risk of pathogen transfer. Had a person with influenza been present on board the *Columbia* steam ship (3 d, 74 passengers) at its time of departure from Oregon, we estimate a 66% risk of introduction into San Francisco (Table 1). By contrast, we estimate just a 1% risk for the *Tarquina* sail ship from Oregon (7 d, 11 passengers), a 0.1% risk on the *Columbus* steam ship from Panama (18 d, 420 passengers) and a 0.1% risk on the *Baltimore* sail ship from Hawai’i (14 d, 29 passengers).

Measles, with longer latent and infectious periods, presents moderate introduction risks across all journey times ≲40 d (Fig. 4*A* and Table 1)—consistent with this pathogen’s range of durations for single-generation outbreaks (Fig. 1*E*) This range includes the vast majority of journeys originating from Oregon (by steam or sail), Hawai’i (by steam or sail), and Panama (by steam). Additionally, we estimate plausible introductions from Panama (by sail) and Hong Kong, especially on ships transporting large populations. Had the *Iowa* sail ship (54 d, 377 people) departed Hong Kong with a measles patient on board, we estimate a 61% chance of introduction despite the long journey (Table 1). Had the *Golden Gate* steam ship (65 d, 458 passengers) departed New York City with one infected passenger, we likewise estimate a 61% introduction risk. Our results are also consistent with reports that the *Sir Charles Napier* sail ship from Panama experienced an outbreak of measles which ended roughly 36 d before arrival; under our base assumptions, we estimate that this vessel had just a 16% chance of sustaining the pathogen across the duration of its 90-d voyage. Supplementary analyses indicate measles introduction following this voyage would have been plausible under some circumstances, particularly if a higher proportion of the population had been susceptible and if shipboard transmission intensity had intermediate intensity (*SI Appendix*, Fig. S1).

Smallpox has a substantially longer generation period than either measles or influenza ($\mu_{E}=12$ d; $\mu_{I}=17.5$ d). Consequently, journeys of ≲50 d present a moderate introduction risk at any ship population size, mirroring the introduction range described above for measles. As before, we estimate higher introduction risks for ships with larger populations (Table 1). Yet since smallpox is less transmissible than measles, ships require larger population sizes to achieve an equivalent $R_{e}\left(0\right)$. Thus, in some cases highly populated ships present a lower risk of introducing smallpox than they did measles; had the *Golden Gate* departed New York City with one infected patient on board, we estimate just a 21% risk of smallpox introduction (Table 1). In practice, by the mid-19th-century widespread smallpox vaccination likely bolstered this effect by reducing $\frac{S(0)}{N}$ relative to other respiratory viruses—although the global extent of vaccination by this time is not yet clear (52).

Our results are consistent with the one documented introduction of smallpox by ship to San Francisco during our period of study (the *Gold Hunter*, a 29-d voyage with 163 passengers). At a susceptibility rate of 5%, we estimate a 42% chance of arriving with at least one active case. Supplementary analyses indicate that introduction risk was in fact highly likely across a wide range of conditions, even including scenarios with no susceptible people on board besides the index case (*SI Appendix*, Fig. S1). This is intuitive; given the pathogen’s lengthy latent and infectious periods (we assume mean values of 12 and 17.5 d, respectively), a single case could easily last as long as the *Gold Hunter*’s period in transit (*SI Appendix*, Table S1).

Finally, we use these analyses to inform the plausibility of ship-borne pathogen transfer across a selection of historical voyages, chosen to reflect the variety of shipping routes, technologies, and practices between the 15th and 20th centuries (Fig. 4*B* and *SI Appendix*, Table S3). For these analyses, we again assume a 5% rate of susceptibility, although in practice we expect this rate varied significantly by location, time period, and ship population.

Under these assumptions, early transatlantic voyages of exploration could plausibly have introduced measles or smallpox to their places of arrival (Fig. 4*B* and Table 2). We estimate a 24% chance of measles introduction and an equal chance of smallpox introduction had Christopher Columbus’s 1492 voyage on *Santa María* (35 d, 41 people) departed with one case of either pathogen on board. We estimate a 2% risk of measles introduction and 4% risk of smallpox introduction on John Cabot’s 1497 exploration on the *Matthew* (54 d, 20 people). Introduction risks for both pathogens were substantially higher on the transatlantic slave trade ship *Diana*, which carried 443 enslaved people and crew from Îles del Los, off the coast of West Africa, to Curaçao, in the Caribbean: a 67% risk for measles and a 35% risk for smallpox, had one person been infected at the time of departure.

**Table 2.** Numerical introduction risk estimates for influenza, measles, and smallpox across selected historical voyages, 1492 to 1918

			Pathogen introduction risk		
Vessel	Days	N	Influenza	Measles	Smallpox
*Santa María*	35	41	<0.001	0.242	0.235
*Matthew*	54	20	<0.001	0.021	0.044
*Sea Venture*	53	150	<0.001	0.318	0.160
*Mayflower*	66	127	<0.001	0.145	0.073
*Diana*	41	443	<0.001	0.667	0.345
*Empress of China*	184	34	<0.001	<0.001	<0.001
*America*	180	175	<0.001	<0.001	0.001
HMAT *Boonah*	17	1,095	0.005	0.905	0.823

Meanwhile, the lengthy journey times of the *Empress of China* trade ship (184 d), which traveled from New York City to present-day Macao, and the *America* convict ship (180 d), which traveled from the United Kingdom to Australia, suggest a compelling explanation for the substantially later introduction of smallpox and measles to the South Pacific. Even with 175 passengers, a voyage such as the *America*’s is outside the range of plausible introduction for all three pathogens.

Our analyses indicate that by far the greatest introduction risk of smallpox and measles—and the only plausible influenza introduction—came from fast, highly populated ships such as the First World War troop ship His Majesty’s Australian Transit *Boonah* (1,095 passengers and crew), here undertaking a 17-d journey from South Africa to Australia. Had this ship departed with one infected person on board, it would have had a 0.5% risk of introducing influenza, an 82% chance of introducing measles and a 91% chance of introducing smallpox to its destination. This combination of fast transit and extremely large passenger populations substantially increased both the magnitude of introduction risk for moderately fast-burning pathogens (such as measles and smallpox) and expanded the range of potential introduction to include pathogens (such as influenza) with much faster life cycles.

## Discussion

Many stories of transoceanic pathogen transfer have focused heavily on early colonial European seafaring. Our analysis indicates that introductions of smallpox and, to a lesser extent, measles from Europe to the Americas via early colonial voyages was plausible, but by no means guaranteed. Depending on weather, these journeys could last just 5 to 10 wk (55), which is a reasonable time frame for these pathogens to persist on board a ship. In these contexts, overall pathogen introduction rates likely depended more on population-side factors—for example, the density of susceptible people, or the rate at which ships departed with active infection(s) on board—than on the precise epidemiological parameters aboard ships. By contrast, our model shows that early transatlantic introductions of faster-burning pathogens such as influenza were unlikely, as were introductions of any acute pathogen on longer journeys such as sail voyages to the Pacific (32).

More recently, the story of transoceanic pathogen transfer has been told as one of technological innovation. Our work supports and extends Cliff and Haggett (33)’s argument that steam technology transformed rates of transoceanic pathogen transfer. Steam ships traveled more quickly, could carry greater numbers of passengers and, in the case of Gold Rush-era San Francisco, made more frequent voyages. Under the right conditions, this could have increased both the rate and the geographic range of transoceanic pathogen transfer substantially (Fig. 4*A* and Table 1).

However, steam travel was not unique in enabling global pathogen circulation. Our analysis confirms longstanding arguments by historians that processes which involved large-scale people-movement—for example war, migration, or the transatlantic slave trade—were enormously significant for global pathogen ecology (8, 9, 56, 57). In the case of 1850s California, ship population size could easily have been the difference between plausible introduction and epidemiological isolation. In 1852, two ships sailed from Hong Kong, the *Catalpa*, and the *Iowa*. Both displayed similar transit times into San Francisco: 60 d and 54 d, respectively (41, 42). Yet while the *Catalpa* carried “Chinese merchandise, rice, cordage, and assorted goods”—along with one solitary passenger—the *Iowa* brought “377 unidentified in steerage,” likely Chinese people bound for California’s gold fields (58). As our analyses show, the presence of 377 people on board transformed the *Iowa*’s capacity to sustain outbreaks of smallpox and measles across the journey from Hong Kong to San Francisco (Fig. 4*A* and Table 1).

Our study specifically considers a small subset of human pathogens, chosen both for their historical impact and because they permit simple modeling approaches. Historical scholarship, together with recent advances in paleogenomic sequencing, demonstrates that transoceanic shipping enabled the diffusion of a much broader range of diseases (12, 59, 60). These include pathogens with food-, water-, and fomite-borne transmission (e.g. cholera, *Salmonella*) (59, 61); pathogens with vector-borne transmission (e.g. malaria, yellow fever, and West Nile virus) (12, 60, 62–64); pathogens with multispecies transmission (e.g. plague, tuberculosis) (60, 65, 66); and pathogens which infected only nonhuman animals (e.g. rinderpest, foot-and-mouth disease) (67, 68). Transoceanic shipping also shaped the global dissemination of broad range of plant and animal species; recent scholarship suggests that these processes were likewise shaped by the speed and volume of transoceanic shipping, as well by trade of specific commodities (69–72). A full understanding of these introductions will require modified modeling approaches and likely additional historical data. This issue is also pertinent to smallpox, for which the extent of fomite transmission is unclear. Recent research indicates that orthopoxviruses can remain viable on surfaces for weeks (73). The World Health Organization’s smallpox eradication campaign found that fomites caused only a small minority of outbreaks (74), but in the context of historical pathogen circulation even rare introductions can be impactful (28–30).

Several additional questions require further consideration. One concerns the mechanics of shipboard transmission. Little is known concerning either the density dependence or the intensity of transmission on board historical vessels (*SI Appendix*, *Text S1*). Our analysis points to several strong qualitative patterns, which are robust across a broad range of parameters. Crowded ships with larger and more susceptible populations presented greater risks regardless of the precise form of density dependence (Fig. 2). Similarly, broad ranges for plausible outbreak duration can be inferred from pathogen natural history, even without knowing shipboard $R_{0}$ (Fig. 1*E*). More refined quantitative predictions will depend on the specifics of particular ships and voyages, and will require further research into shipboard transmission dynamics. We have used our model to map the possible effects of assumptions regarding density dependence and transmission intensity across a range of plausible parameters (*SI Appendix*, Figs. S1–S4).

A second question concerns the extent of shipboard population structure. Ships were famously hierarchical environments, and highly compartmentalized populations may have prolonged ship outbreaks. Captains or surgeons may also have manipulated population structure in response to outbreaks, for example through case isolation, quarantine, or disembarkation of known or suspected infections. While population structure almost certainly shaped outbreak duration, incorporating these effects is challenging in the absence of high-resolution outbreak data from a given ship. Moreover, on ships with poor ventilation or hygiene practices, transmission could plausibly have been homogeneous regardless of social behaviors or most medical interventions.

Our work sheds light on how shipboard transmission dynamics shape introduction risk, but reconstructing historically accurate circulation rates would require more information regarding pathogen dynamics in source populations. This matters for inferring likely immunity rates in ship populations, which our model shows can have a large impact on estimated risks (*SI Appendix*, Fig. S5). It also matters for assessing the probability of at least one infected individual on board ship at the point of departure. Longitudinal mortality data exist for diseases such as smallpox and measles, especially in European and North American contexts, with particularly well-preserved time series in the London Bills of Mortality (27, 75). Reconstructing historical prevalence and immunity landscapes from these sources is difficult, but is critical for estimating realistic pathogen transfer rates pre-20th century contexts.

A related question concerns the contribution of partly immune individuals to pathogen circulation within a given population. In 19th-century contexts, this could include either waning natural immunity or, in the case of smallpox, waning vaccinal protection (74). The ability of partly immune people to be infected and transmit infection has long been recognized as an important driver of influenza (76) and smallpox (74) epidemiology. Partial immunity also provides a compelling explanation for recent resurgences in mumps (77) and pertussis (78, 79). Moreover, it is plausible that the contribution to transmission from partly immune individuals was more significant on board a ship than it was on land, due to extended exposures or large infectious doses. This possibility—and the influence of partial immunity on outbreak duration more broadly—require further investigation.

Our model offers a general assessment of outbreak duration in a closed population, which holds significance beyond historical systems. Understanding infection persistence in discrete subpopulations is critical for studying pathogen circulation in any system with limited host connectivity, from wildlife populations (80, 81) to agricultural biosecurity (82, 83) to human populations distributed across regions (17, 84).

These findings carry important historical implications, connect to present-day disease dynamics, and may, some day, inform interplanetary risk of pathogen spread. Centuries before the present-day upheaval of air travel (84–86), technological innovation and large-scale human relocations combined to transform global pathogen ecology (87). Yet this process was almost certainly lengthy, geographically uneven, and contingent on complex interplays between technology, shipping practices, and specific pathogen biology. This presents a rich avenue for collaboration between ecologists, epidemiologists, historians, and social scientists. How do social, economic, and technological forces combine to shape global pathogen ecology—and with what consequences along the way for the world’s people, places, and pathogens?

## Materials and Methods

We simulate shipboard outbreaks using a stochastic Susceptible-Infectious-Exposed-Recovered (SEIR) model (*SI Appendix*, *Text S3*). We implement continuous-time stochastic simulations in R with the Gillespie Algorithm, using the package GillespieSSA (88). All simulations assume a single index case in state E at the time of departure. We define outbreak duration as the time until both state E and state I contain zero individuals.

To achieve a more realistic depiction of the time course of infection, we use the Linear Chain Trick to make dwell times in state E and state I Erlang-distributed (89). For all simulations, we use shapes $k_{E}=k_{I}=3$ and rates $k_{E}/\mu_{E}$ and $k_{I}/\mu_{I}$ for states E and I respectively. This technique gives a unimodal distribution with a long right-hand tail, such that disease progression is relatively constrained in most individuals, but occasional individuals experience substantially longer periods of incubation or infectiousness (90). We assume that state E is presymptomatic and that initially infected individuals could board ship at any point during this period, randomly assigning index cases across substates $E_{1},E_{2},...,E_{k_{E}}$ at the point of departure.

Our model also tracks infection across pathogen generations, when needed. The $I_{n}$ infectious individuals from generation n produce new exposed individuals $E_{n+1}$, which represent the ${\left(n+1\right)}^{\mathrm{st}}$ generation of infections.

To account for uncertainty and variation in the density dependence of shipboard contact rates, our model uses a flexible depiction of density dependence encoded by the equation:

$$R_{0}=\mu_{I}{\left(\beta_{\mathrm{dd}}N\right)}^{q}{\left(\beta_{\mathrm{fd}}\right)}^{1-q},$$

$R_{0}$ is the pathogen’s basic reproduction number on board a given ship. This represents the average number of infections that an infected person generates in a fully susceptible population, where $\mu_{I}$ is the average period of infectiousness. The density dependence of transmission is adjusted with the parameter q, with q=1 representing classical density-dependent transmission ($R_{0}\propto N$), q=0 representing classical frequency-dependent transmission ($R_{0}╨\;N$), and 0<q<1 representing intermediate density dependence ($R_{0}\propto N^{q}$). The parameters $\beta_{\mathrm{dd}}$ and $\beta_{\mathrm{fd}}$ modulate the intensity of transmission under each density dependence pole—-intuitively, the proportion ($\beta_{\mathrm{dd}}$) and the raw number ($\beta_{\mathrm{fd}}$) of people on board ship that a single infected individual will infect per day, on average, in a fully susceptible population.

In analyses where N is constant and where we do not explore the effect of density dependence, we set q=1 such that $R_{0}=\beta_{\mathrm{dd}}N$; we then backcalculate $\beta_{\mathrm{dd}}$ from $R_{0}$ and N. Mathematically, this is equivalent to setting q=0 with $\beta_{\mathrm{fd}}$ fixed at $\beta_{\mathrm{fd}}=\beta_{\mathrm{dd}}N$.

For analyses where N varies, we infer $\beta_{\mathrm{fd}}$ from literature values of $R_{0}$ and $\mu_{I}$ (*SI Appendix*, Table S1) and set $\beta_{\mathrm{dd}}=\beta_{\mathrm{fd}}/c$. Here, c is a constant representing the population size at which $\beta_{\mathrm{dd}}$ and $\beta_{\mathrm{fd}}$ would be equal. In main text analyses, we set c=100. We explore alternative values of c, as well as a range of q values, in *SI Appendix*, Figs. S2–S4.

### Historical Data.

To provide real-life context for our theoretical results, we collected data on ship arrivals into the port of San Francisco between June 6, 1850, and June 19, 1852, from volumes I, II, and III of genealogist and historian Louis. J. Rasmussen’s reference book *San Francisco Ship Passenger Lists* (41–43). We recorded the port of origin, the ship type, the journey time, and the number of passengers for ships originating from seven locations: Hawai’i; Hong Kong; Oregon Territory; New York City; Sydney, Australia; Valparaíso, Chile; and Liverpool, England. In the few cases where Rasmussen reports ships making multiple stops at one or more of these locations in the course of their voyage, we record both the journey time into San Francisco from a ship’s port of origin and, where available, journey times into San Francisco from the intermediate port(s). We exclude ships where substantial numbers of people (N>10) boarded during a voyage, as our model does not account for changes in population size subsequent to the initial point of departure.

For almost all ships, Rasmussen provides passenger numbers but not numbers of crew. We assume that in most cases, crew i) represented a small proportion of a ship’s total population; ii) were, as professional sailors, more likely to possess immunity to common maritime infections, and so represented an even smaller proportion of a ship’s susceptible people. Thus, in the absence of crew size data, analyses considering population size of vessels arriving into San Francisco approximate N as the total number of passengers on board each ship.

## Supplementary Material

Appendix 01 (PDF)

## Data Availability

Previously published data were used for this work (41–43).
