# Supplementary material for: Transoceanic pathogen transfer in the age of sail and steam
Source: Proc Natl Acad Sci U S A. 2024 Jul 16;121(30):e2400425121. doi: 10.1073/pnas.2400425121 (PMC11287167; doi:10.1073/pnas.2400425121)
Supplement: Supplementary file 1 — Appendix 01 (PDF) [file pnas.2400425121.sapp.pdf]

Supporting Information for

Transoceanic Pathogen Transfer in the Age of Sail and Steam

Elizabeth N. Blackmore and James O. Lloyd-Smith

James O. Lloyd-Smith
E-mail: jlloydsmith@ucla.edu

This PDF file includes:

Supporting text
Figs. S1 to S5
Tables S1 to S4
SI References

Supporting Information Text

1. Transmission on board historical ships

A. Historical evidence. We expect that transmission on board historical ships was substantially more intense than transmission in land settings. In a study of pandemic 1918 influenza, Vynnycky et al. (1) estimated R_0 values of approximately 4-11 on board the troop ship His Majesty's Australian Transport (HMAT) *Boonah*, approximately 5-17 on board the troop ship HMAT *Devon*, and approximately 3.5-8 on board the troop ship HMAT *Medic*. These compare to an estimated range of roughly 1.5-4 in American and Scandinavian towns and cities. Using a different method, White and Pagano (2) calculated an R_0 of roughly 4.97 for the same epidemics on board HMAT *Boonah* and HMAT *Medic*, compared with an R_0 of 1.34-3.21 in Maryland communities.

Similar analyses are not available for earlier time periods. However qualitative descriptions likewise indicate that conditions on board pre-1918 ships were highly conducive to intense pathogen transmission. One 1801 newspaper report describes an emigrant ship from Ireland to New York City so crowded that "the space between decks, occupied by nearly 300 persons, became the receptacle of all excremental matters". (3). Half a century later, English author Frank Marryat recalled travelling from Panama to San Francisco on a boat "so crowded with passengers, that it was not until it was ascertained that there was scarcely standing-room for those on board that she tripped her anchor" (4). Longer excerpts from both texts, printed below, offer vivid depictions of the conditions that passengers suffered during these voyages.

In addition to crowding, pre-1918 ships also faced substantial ventilation challenges. On sail ships, opening portholes risked losing heat and letting in water. Thus, below-deck spaces were sealed, resulting in notoriously poor air quality (5, 6). The steam revolution somewhat improved onboard environments, both by providing a source of heat and, in some cases, powering active ventilation systems. Yet steam ventilation still failed – sometimes catastrophically. In 1848, seventy-three people died from suffocation on board the *Londonderry* emigrant steamer when hatches were closed during a storm (6). Six years later, fifty passengers out of a total of 204 died by suffocation in an unnamed ship carrying emigrants from Mauritius (7). "Under no circumstance can a ship of any kind be made as healthful or as comfortable as the house on shore," wrote United States Navy surgeon Albert L. Gihon in 1886. Gihon added: "It is, practically, a floating box sealed against the admission of water, and, of course, also of air." (8)

B. Density dependence. The nature of density dependence of pathogen transmission on board historical ships is unclear. On land, early-twentieth century studies demonstrate a clear and intuitive link between population density and rates of respiratory infection. Notably, Brewer (9) documented higher rates of pandemic influenza in military units training in more crowded environments in a 1918 study of Fort Humphreys, Virginia.

Some contemporaries clearly thought of ship transmission in what we would today consider density-dependent terms. In a report on 1918 pandemic influenza on board Royal Navy ships, surgeon-commander Sheldon F. Dudley argued that "infective material must become so dense and diffused as to saturate the ship. That is to say, everyone on board receives a dose of the specific agent sufficient to cause influenza" (10). Dudley's report is excerpted below.

Yet density dependence does not appear to describe every instance of shipboard transmission. In an analysis of smallpox outbreaks on board vessels bound into Australia, 1850-1908, quarantine director J. H. Cumpston found that most shipboard smallpox transmission was limited to close contacts of infected patients, such as family members, close colleagues, or those sharing beds and cabins (11). Such a pattern typically argues for "frequency-dependent" transmission, in which an infected individual on average transmits only to a fixed number of close contacts, regardless of a ship's total population size.

C. Contemporary examples. Even with the benefits of modern hygiene, sophisticated air circulation, and more highly-regulated living conditions, infectious disease outbreaks are common on present-day cruise (12–14), cargo (15) and naval ships (16). Attack rates are often high. Vera et al. (17) reported a 49.1% attack rate of 2009 H1N1 pandemic influenza in an unnamed Peruvian Navy ship carrying 355 people, with greatest risk in cadets assigned high-density living quarters. Earhart et al. (18) observed a 42% attack rate of H3N2 influenza across more than 500 people on board Navy vessel USS *Arkansas*, despite 95% vaccination coverage. Brotherton et al. (2000) reported a 37% attack rate of influenza-like illness on a cruise ship carrying over 1600 passengers and crew.

Recent reports of SARS-CoV-2 transmission on board passenger ships (19–22) and military ships (23) indicate high attack rates across both vaccinated and unvaccinated populations. Multiple studies suggest that outbreaks could spread broadly across ship populations. Using a mechanistic model, Azimi et al. (19) estimate similar transmission contributions from long-range aerosols and from short-range aerosols and droplets on board the *Diamond Princess* cruise ship. Meanwhile, in a study of an extensive outbreak on board a Dutch river cruise (60 of 132 passengers infected), Veenstra et al. (22) observed no apparent clustering by cabin layout or by meal-time seating arrangements.

D. Density dependence parameter choices for historical analyses. Given the limited quantitative studies of density dependence on board historical ships, parameter choices for the analyses supporting Figure 4, Table 1, and Table 2 are necessarily approximations. We assume intermediate density-dependence ($q = 0.5$) and infer β_{fd} from standard literature values for R_0 and for each pathogen's infectious period, μ_I ($\beta_{fd} = R_0/\mu_I$) (Table S1). We set $\beta_{dd} = \beta_{fd}/c$, where c intuitively represents the value of N at which β_{fd} and β_{dd} would be equal. Using the general expression $R_0 = \mu_I(\beta_{dd}N)^q(\beta_{fd})^{1-q}$, and substituting $\beta_{dd} = \beta_{fd}/c$, we obtain $R_0 = \mu_I\beta_{fd}(N/c)^q$. Thus for our assumption of $q = 0.5$ on a ship, the ratio of shipboard R_0 to frequency-dependent transmission ($R_0 = \mu_I\beta_{fd}$) is $(N/c)^{0.5}$.

To choose an appropriate value of c, we referred to the analyses of shipborne R_0 by Vynnycky et al. (1) and White and Pagano (2), discussed above. In ship settings, Vynnycky et al. (1) estimate an R_0 of $\sim$4–17 for 1918 influenza on three $\sim$1,000-person ships, compared with an R_0 of 3.5–8 on land. White and Pagano (2) re-analysed two of the three ships in Vynnycky et al.'s analysis and found an R_0 of $\sim$4.97, compared with an R_0 of 1.34–3.21 on land. Altogether, these analyses suggest that R_0 for influenza on board a 1,000-person ship is roughly 2.5- to 4.5-fold greater than its value on land. Assuming that these land R_0 values represent frequency-dependent transmission, and assuming intermediate density dependence ($q = 0.5$) on board the three ships in these analyses, we back-calculate a c range of roughly 50–150. We use $c = 100$ in main text analyses. We explore additional values ($c = 50, c = 150$) in supplementary figures S2–S4, as well as a broader range of q values.

2. Qualitative Descriptions of Infection and Transmission in Shipboard Settings, 1801-1919

Three written descriptions of disease transmission on board nineteenth- and early twentieth-century ships are excerpted below. We do not intend these extracts to give a comprehensive view of shipboard transmission, but rather to offer a qualitative view of possible transmission scenarios. All spelling and grammar is original.

A. "Another instance of pestilence engendered in a ship crowded with passengers from Ireland.". (3)

The ship Nancy, Capt. John Herron, was charterd by a commercial house at Sligo, to carry passengers from that port to New-York. She sailed from Sligo on the 12th July, 1801, and arrived, after a passage of 77 days, at the port of New York, on the 27th of September following. This ship, of the burthen of 202 tons, received on board 417 passengers, and was navigated by nine seamen. The provisions, mere refuse, put up by government-contractors with the view of saving expense, were of the worst kind: and the water, which was also of bad quality, from the unexpected length of the voyage, became extremely scanty before the arrival of the ship.

In order to receive so great a number of passengers on board of this ship, temporary cabbins [sic] were built on the quarter-deck, which were filled with eighty persons. Three hundred were crowded into the space between decks.

It will excite no surprise that a vessel thus crowded became sickly soon after sailing from Sligo. Typhous fever and dysentery began to prevail, and destroyed the lives of a large proportion of the passengers.

In addition to the wretchedness of being confined in so small a space, these unhappy emigrants suffered all the evils which their habits of uncleanliness could produce. Their bodies and clothes, covered and saturated with filth, exhuded poison all around them. Partly from the want of strength and assistance among the sick, and partly from the want of a sense of decency, the space between decks, occupied by nearly 300 persons, became the receptacle of all excremental matters, insomuch that they issued in streams from the scuppers. The filth on the upper deck was nearly over the shoes. The sides of the ship were daubed and incrusted [sic] with excrements; and even the rope for the support of such that wished to go on board were unfit to be handled. The stench was intolerably offensive.

In such condition arrived this unfortunate vessel at the place assigned for quarantine in the port of New York. Ninety persons had died on the passage; one hundred and eighty were sick. Scarcely a healthy countenance was to be seen on board of the ship; very few had escaped disease; and many had suffered from three to four relapses. About forty were taken ill after their arrival.

As soon as possible after their arrival the sick were brought ashore; stropped of their filthy and pestilential clothes; their bodies thoroughly washed and scoured with soap and water; and then wrapped up in clean blankets, and carried into the wards appointed for their reception in the Marine Hospital. The permanent buildings of the establishment were insufficient to receive so great a number; tents, and other temporary accommodations, were provided for the remainder. Separation, ventilation, and cleanliness, as soon as they could be brought into action, accomplished every thing that could be expected. And only twenty-six have died since their arrival at this port.

B. Frank Marryat's description of a voyage from Panama to San Francisco, 1851. (4)

It seemed that we had brought the yellow fever with us to Panama, or rather it appeared at the time of our arrival, and it was now spreading with great rapidity. Cholera also broke out, and deaths from one or the other of these causes became very numerous.

The people being panic-struck, a great rush was made for the Californian boats, of which there happened, at this time, to be very few.

So soon as I was able to move, there was but one small screw steamer in port, and as the place was daily becoming more unhealthy, I secured, by great favour, a cabin in her.

Nothing could excuse the state in which this ship was put to sea, not even the panic; for she was not only ill-found in every respect, but was so crowded with passengers, that it was not until it was ascertained that there was scarcely standing-room for those on board that she tripped her anchor.

I had secured a dog-hole of a cabin, and was no sooner on board than my wife, worn out by fatigue and anxiety, was attacked by violent fever. There were two young doctors on board, but both were attacked shortly after we started. Then the epidemic (an aggravated intermittent fever) broke out among the passengers, who – crowded in the hold as thick as blacks in a slaver – gave way to fear, and could not be moved from the lower deck, and so lay weltering in their filth.

C. Sheldon F. Dudley's report of 1918 pandemic influenza on Royal Navy vessels, 1921. (10)

The density of susceptible persons in a ship must be very great as compared with an assemblage of susceptible persons on shore. If, for example, we contrast the sleeping accommodation in a ship with that of a big institution, we find that in the ship

hammock hooks are less than 2 ft. apart, whereas institution bed centres are rarely less than 8ft.apart. Even when head to toe slinging is insisted on in a ship the men's heads must often be within 3ft. of each other. Now the volume of spray from a mouth at 3ft. is nearly twenty times that at 8ft. Therefore how much more readily will the man sleeping in a battleship's mess-deck get a requisite dose of infectious material than the man sleeping in an institution ashore? Again we also hear of many men in a ship not ill enough to go off duty, and, thus being immobilized, wandering about among their fellows.

When we consider these points, and at the same time realize that a modern battleship, with its tiers of lumbered decks, its cramped accommodation, and its crew of often over 1,000 men, covers an area of less than one-fiftieth of a square mile, 1 do not think it possible to doubt the infective material must become so dense and diffused as to saturate the ship. That is to say, everyone on board receives a dose of the specific agent sufficient to cause influenza, unless he happens to be highly immune at that time to the strain of organism responsible for the epidemic. In a ship, the density of susceptible persons, the mass of infection, and the local migration are all so great that any diminution in one or more of these factors that may be produced by the use of sprays and gargles, by early isolation of cases and disinfection, is scarcely likely to diminish the rate of spread in a ship, once influenza has obtained a footing on board. And I think naval experience, where all these preventive measures have been vigorously employed, justifies this pessimism, as I have been unable to learn of any definite cases in which they did any good. In ships the outbreaks lasted ten days to three weeks; ashore the wave took about three months to pass over a locality. The longer wave period ashore was probably due to the lesser density of susceptible persons and infective sources, more time being required for the infection to hunt out all the susceptible individuals within its reach.

3. Model Equations

To achieve a more realistic depiction of the time course of infection, we make dwell times in state E and state I Erlang-distributed using the Linear Chain Trick (24). This technique gives a unimodal distribution with a long right-hand tail, such that disease progression is relatively constrained in most individuals, but with occasional individuals experiencing substantially longer periods of incubation or infectiousness (25).

Individuals progress through k_E exposed states, $E_1, E_2, ..., E_{k_E}$, and through k_I infectious states, $I_1, I_2, ..., I_{k_I}$. We use $k_E = k_I = 3$ for all simulations.

The rate of progression from state E_e to state E_{e+1} and from state E_{k_E} to state I_1 is $\frac{k_E}{\mu_E}$, where μ_E represents the mean length of time that an individual spends in all exposed states. Similarly, the rate of progression from state I_i to state I_{i+1} and from state I_{k_I} to state R is is $\frac{k_I}{\mu_I}$, where μ_I represents the mean length of time that an individual spends in all infectious states.

We track infection across $g > 1$ transmission generations, where $E_{n,e}$ and $I_{n,i}$ denote n^{th}-generation individuals in states E_e and I_i respectively. The $\sum_{i=1}^{k_I} I_{n,i}$ infectious individuals from generation n produce new exposed individuals $E_{n+1,1}$, which represent the $(n+1)^{\text{st}}$ generation of infections.

The number of first-generation individuals, $n = 1$, is fixed at $t = 0$. For all simulations, we assume a single first-generation individual. We randomly assign this person to a state from $E_{1,1}, E_{1,2}, ...E_{1,k_E}$ at the time of departure, $t = 0$.

To account for uncertainty and variation in the density dependence of shipboard contact rates, our model does not assume either classical density dependence or classical frequency dependence. Instead, we model the shipboard transmission with the equations:

$$\frac{dS}{dt} = -\beta_{fd}^{1-q}(\beta_{dd}N)^q S \sum_{n=1}^{g}\sum_{i=1}^{k_I}\frac{I_{n,i}}{N} \qquad 0 \le q \le 1$$

Here, q represents the degree of density dependence on board the ship, with $q = 0$ representing classical frequency dependence, $q = 1$ representing classical density dependence, and $0 < q < 1$ representing intermediate modes of transmission. The parameters β_{dd} and β_{fd} modulate the intensity of transmission under each density dependence pole — intuitively, the proportion (β_{dd}) and the raw number (β_{fd}) of people on board ship that a single infected individual will infect per day, on average, in a fully-susceptible population. Since susceptible people can be infected by infectious people in any generation and at any stage of infection, $\frac{dS}{dt}$ is proportional to the total number of infected people across all g transmission generations and all k_I infectious states.

The following equations show the deterministic analogue of our model. We implement continuous stochastic simulations in R using the Gillespie Algorithm, using the package GillespieSSA (26).

$$\begin{aligned}
\frac{dS}{dt} &= -\beta_{fd}^{1-q}(\beta_{dd}N)^q S \sum_{m=1}^{g}\sum_{j=1}^{k_I}\frac{I_{m,j}}{N} \\
\frac{dE_{1,1}}{dt} &= -\frac{k_E}{\mu_E}E_{1,1} \\
\frac{dE_{n,1}}{dt} &= \beta_{fd}^{1-q}(\beta_{dd}N)^q S \sum_{j=1}^{k_I}\frac{I_{n-1,j}}{N} - \frac{k_E}{\mu_E}E_{n,1} && 2 \le n \le g \\
\frac{dE_{n,e}}{dt} &= \frac{k_E}{\mu_E}(E_{n,e-1} - E_{n,e}) && 1 < n \le g;\ 2 \le e \le k_E \\
\frac{dI_{n,1}}{dt} &= \frac{k_E}{\mu_E}E_{n,k_E} - \frac{k_I}{\mu_I}I_{n,1} && 1 \le n \le g \\
\frac{dI_{n,i}}{dt} &= \frac{k_I}{\mu_I}(I_{n,i-1} - I_{n,i}) && 1 \le n \le g;\ 1 < i \le k_I \\
\frac{dR}{dt} &= \frac{k_I}{\mu_I}\sum_{m=1}^{g} I_{m,k_I}
\end{aligned}$$

169 ## 4. Supplementary Figures

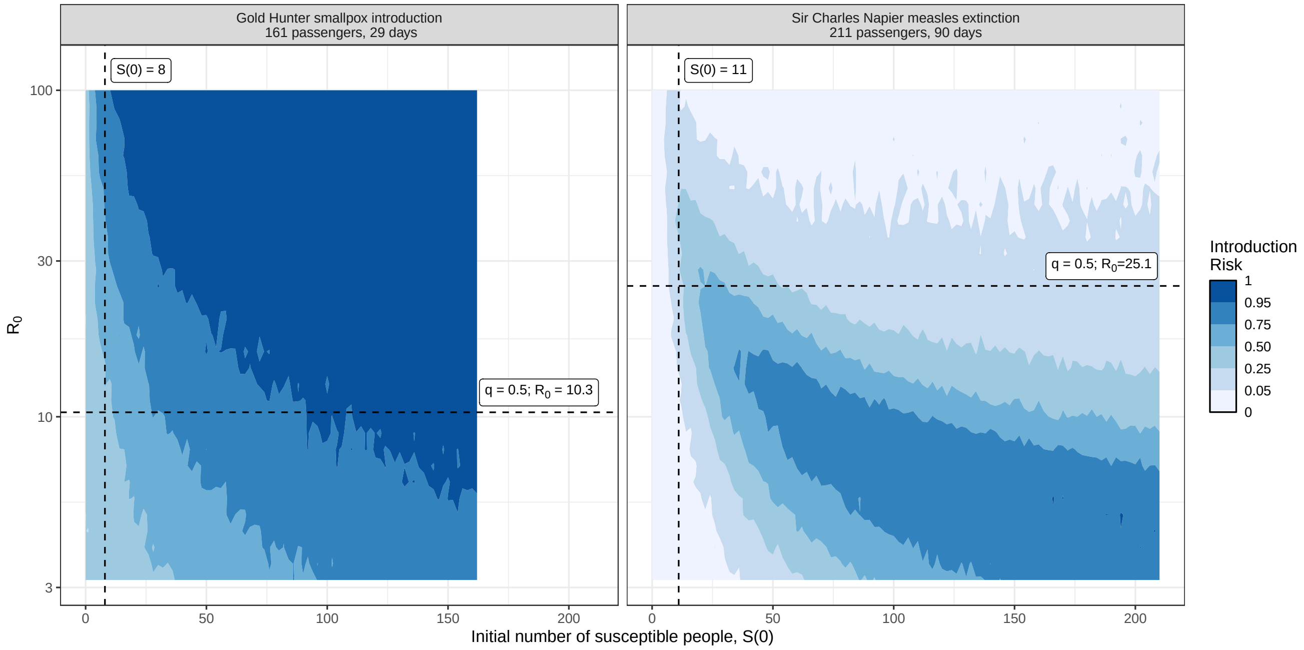

Fig. S1. Introduction risk for documented pathogen outbreaks on board the *Gold Hunter* (smallpox) and the *Sir Charles Napier* (measles) by initial number of susceptibles, $\mathbf{S(0)}$ and by transmission rate, $\mathbf{R_0}$. All analyses assume a single initial index case ($e_0 = 1$). X-axis limits reflect each ship's population: 163 for the *Gold Hunter* and 211 for the *Sir Charles Napier* (Table S4). We use limits of [0,162] and [0,210] for the *Gold Hunter* and the *Sir Charles Napier*, respectively, where 0 represents no susceptible people other than the index case. Introduction probability represents the likelihood of sustained outbreaks across a journey of 29 days (*Gold Hunter*) and 90 days (*Sir Charles Napier*). Horizontal and vertical lines reflect, respectively, the values of $S(0)$ and R_0 used in Figure 4, Table 1, and Table 2.

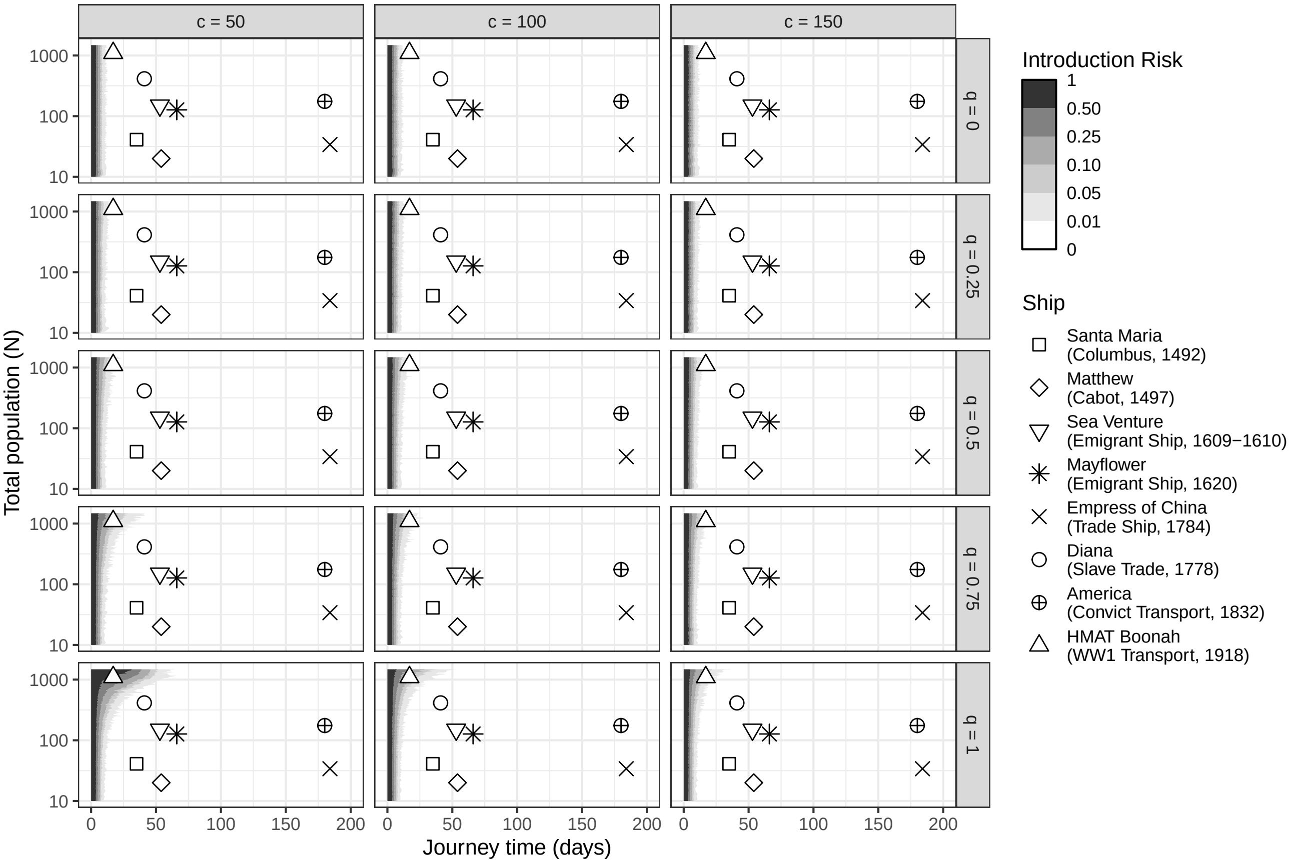

Fig. S2. Sensitivity of influenza introduction risk to density dependence index, q, and to density dependence scaling constant, c. All analyses assume a single index case ($e_0 = 1$), a population susceptibility rate $S(0)/N = 0.05$, and natural history parameters μ_E, μ_I and β_{fd} as given in Table S1.

.

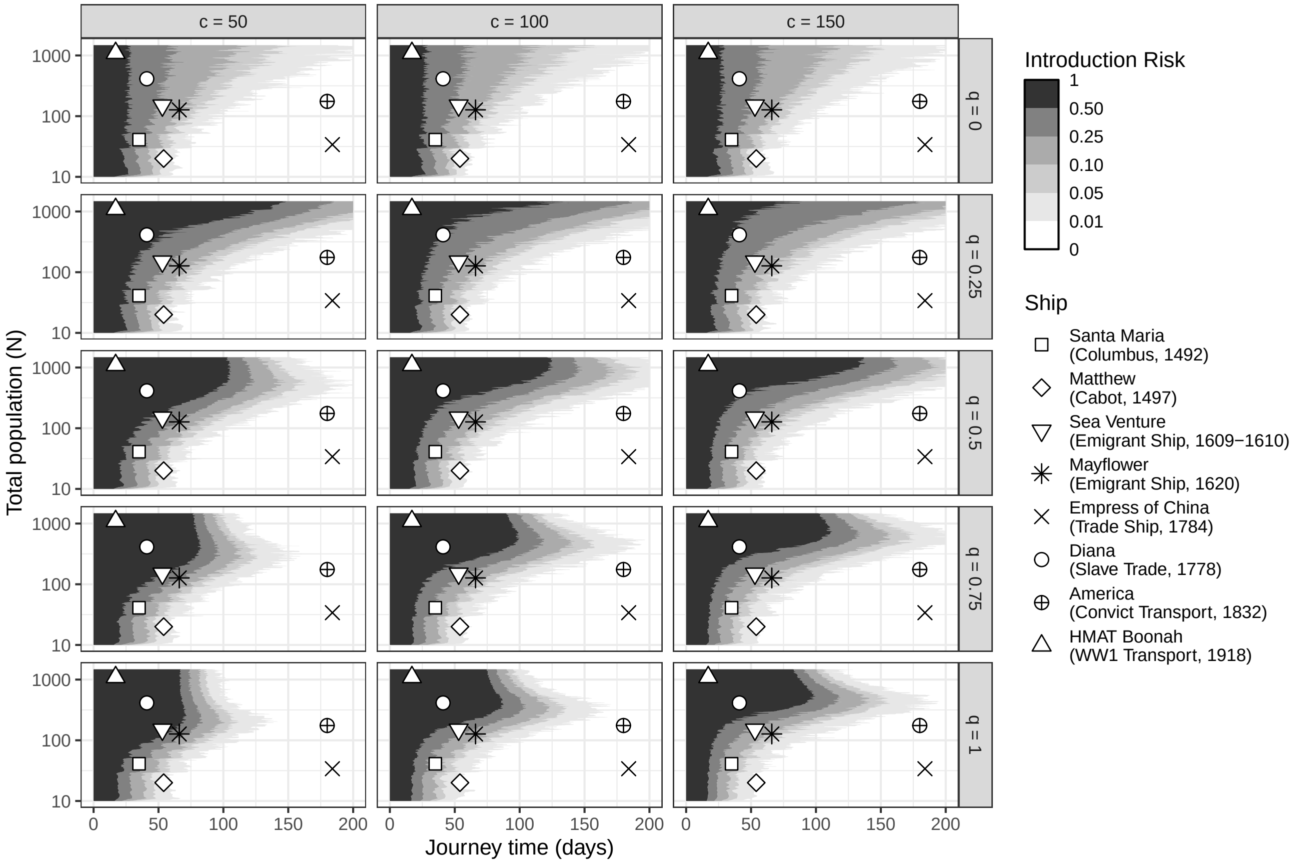

Fig. S3. Sensitivity of measles introduction risk to density dependence index, q, and to density dependence scaling constant, c. All analyses assume a single index case ($e_0 = 1$), a population susceptibility rate $S(0)/N = 0.05$, and natural history parameters μ_E, μ_I and β_{fd} as given in Table S1.

.

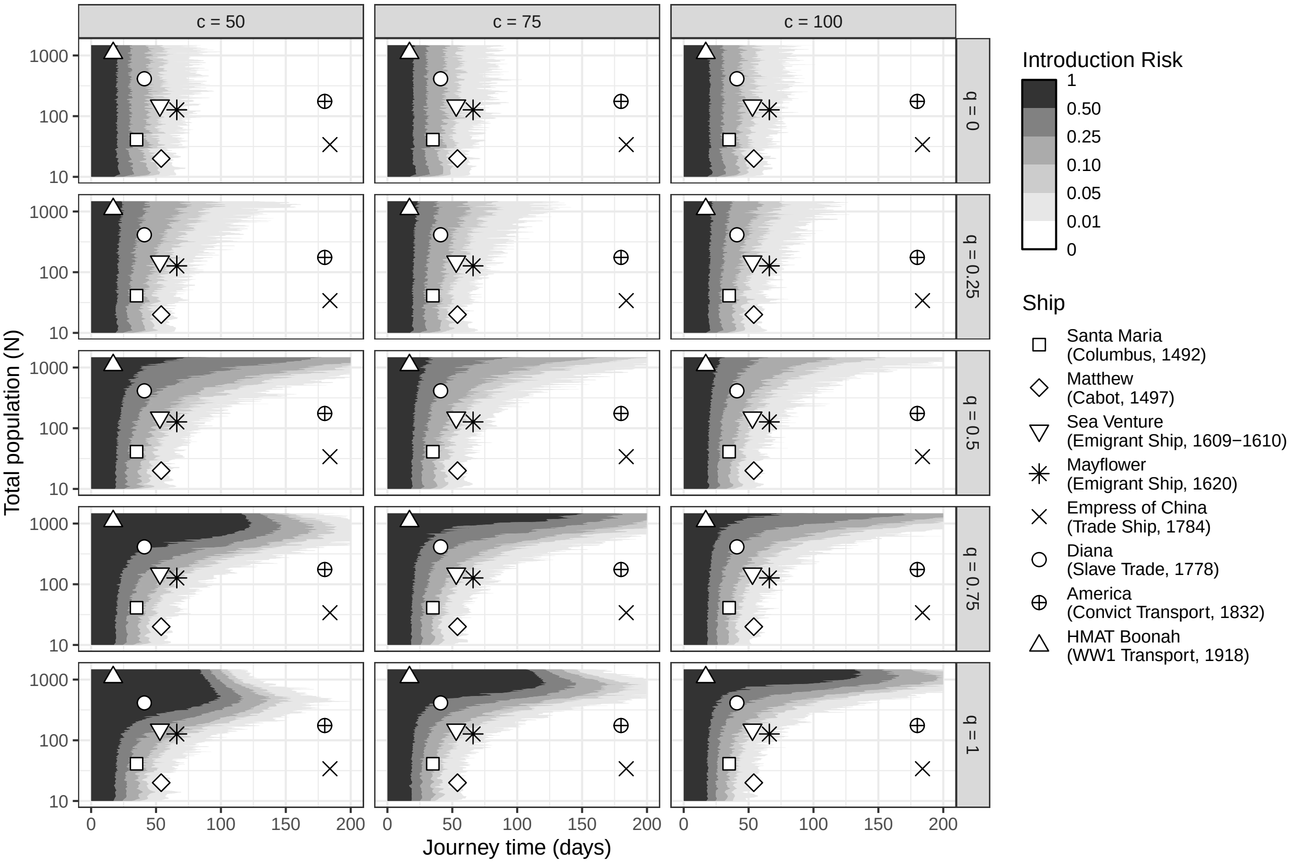

Fig. S4. Sensitivity of smallpox introduction risk to density dependence index, q, and to density dependence scaling constant, c. All analyses assume a single index case ($e_0 = 1$), a population susceptibility rate $S(0)/N = 0.05$, and natural history parameters μ_E, μ_I and β_{fd} as given in Table S1.

.

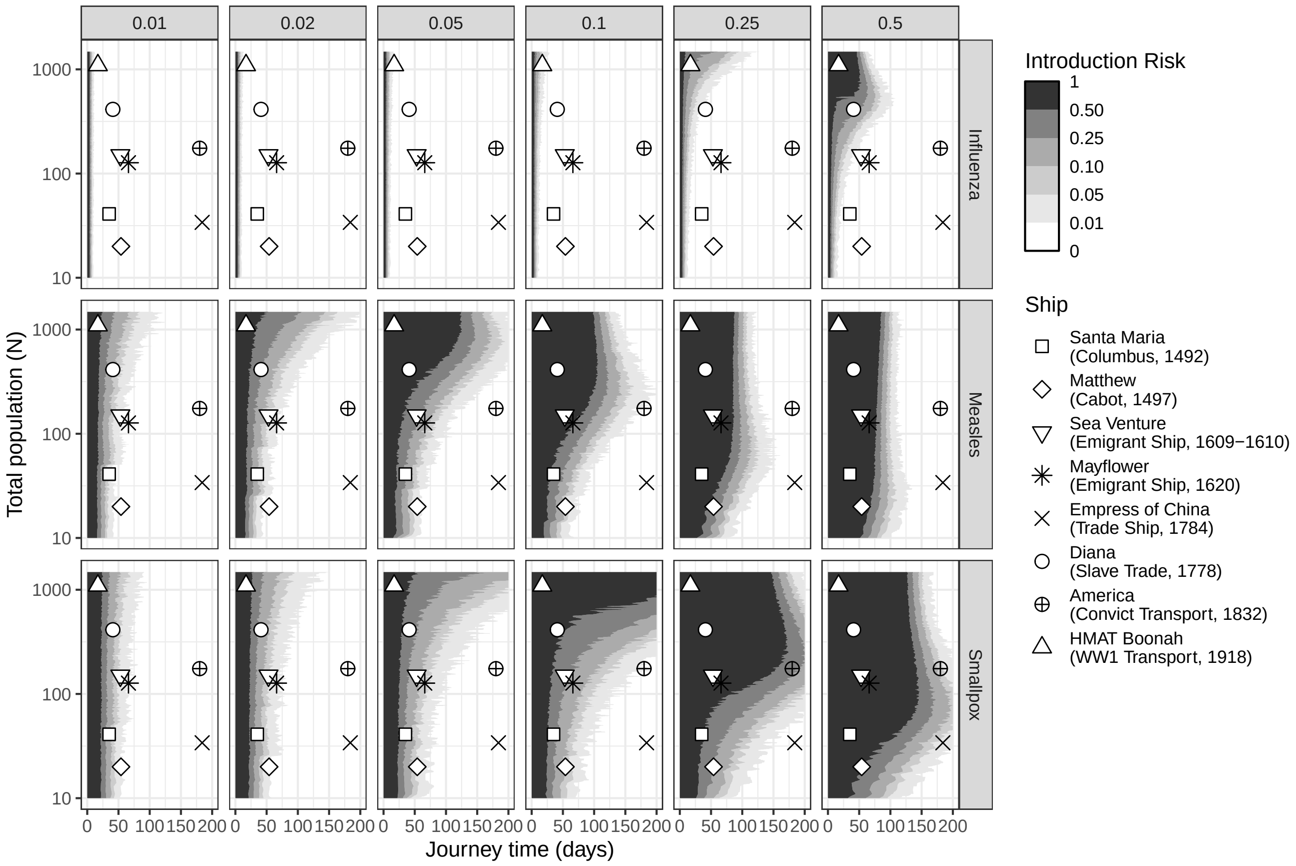

Fig. S5. Sensitivity of introduction risk to initial population susceptibility, $S(0)/N$, by pathogen. All analyses assume a single index case ($e_0 = 1$), intermediate density dependence ($q = 0.5$), a density dependence scaling constant $c = 100$, and pathogen-specific natural history parameters as given in Table S1.

Table S1. Natural History Parameters

Pathogen	Mean latent period (days)	Mean infectious period (days)	Typical Land R_0	β_{fd} used for Figure 4, Tables 1–2	β_{dd} used for Figure 4, Tables 1–2	R_0 used for Figure 4, Tables 1–2	Reference
Influenza	2	3	1.5	0.5	0.005	$0.05\sqrt{N}$	(27–29)
Measles	12	8	15	1.875	0.01875	$0.1875\sqrt{N}$	(27, 30)
Smallpox	12	17.5	7	0.4	0.004	$0.04\sqrt{N}$	(31–33)

Table S2. San Francisco Port Arrivals Statistics, 6 June 1850 to 19 June 1852

			Journey Time			Number of Passengers		
Origin	Type	*n*	Median	Range	Interdecile Range	Median	Range	Interdecile Range
Liverpool, UK	Sail	18	189.5 days	150-300 days	162-245.5 days	4.5	1-18	2-18
New York City, USA*	Sail	92	155.5 days	89-283 days	113.1-219.8 days	5	0-160	1.1-20.0
	Steam	9	180 days	65-364 days	87.2-296 days	111	5-743	11.4-515.0
Valparaíso, Chile	Sail	100	57 days	23-190 days	45.0-74.2 days	7	0-175	1.0-39
Panama**	Sail	39	63 days	36-122 days	43.4-83.4 days	53	1-287	4.6-164.6
	Steam	116	20 days	12-58 days	15.7-28.0 days	196	11-1050	50.6-527.4
Oregon	Sail	48	7 days	3-16 days	3.4-11.3 days	4	0-12	1.0-6.0
	Steam	47	3 days	2-8 days	2.5-5.0 days	28	3-157	12.0-60.0
Hawai'i	Sail	76	22.5 days	14-80 days	16.0-31.5 days	5	0-142	2.0-22.0
	Steam	1	13 days	–	–	18	–	–
Sydney, Australia	Sail	20	91 days	67-188 days	70.0-111.2 days	15.5	2-142	3.9-57.0
Hong Kong	Sail	14	58.5 days	33-95 days	46.7-84.0 days	201	1-553	14.5-378.4

* For reasons that are sometimes unclear, four steam voyages originating in New York City had transit times longer than 200 days: the *SS New Orleans* (210 days), the *SS Sea Bird* (240 days), the *SS Goliah* (279 days) and the *SS Chesapeake* (364 days). The *SS Sea Bird* ran aground on San Martine on its way to California and had to be repaired (34). Meanwhile, the extraordinarily long voyage of the *SS Chesapeake* astonished contemporary observers, with companies who had shipped goods on the vessel suing for damages on its arrival (35).

** Voyages from Panama predate the Panama Canal, which opened in 1914. Prior to the canal's construction, travellers from Europe and from eastern North America took a ship to the east coast of Panama, crossed the Panama Isthmus by land, and took a second ship from Panama's west coast to San Francisco (36).

Table S3. Selected Historical Voyages, 1492-1918

Year	Vessel	Type	Purpose	Journey	Duration	Population	Reference
1492	*Santa María* [Columbus's Second Voyage]	Sail	Exploration	San Sebastian de la Gomera, Canary Islands to San Salvador, present-day Bahamas	35 days	41-60 crew (estimates vary; we use the more recent estimate of 41)	(37)
1497-98	*Matthew* [Cabot's Second Voyage]	Sail	Exploration	Bristol, England to a disputed location in northeastern North America, possibly present-day Maine, Newfoundland, or Nova Scotia	54 days	20 crew	(38)
1609	*Sea Venture*	Sail	Emigration	Bristol, England to present-day Bermuda	53 days	150 passengers and crew	(39)
1620	*Mayflower*	Sail	Emigration	Leiden, Holland to near Cape Cod, Massachusetts	66 days	102 passengers, 20-30 crew (we assume 25 crew)	(40)
1784	*Empress of China*	Sail	Trade	New York City to Macao	184 days	34 crew	(31)
1778	*Diana*	Sail	Slave ship	Iles de Los, present-day Guinea to Curaçao	41 days	413 enslaved people, 30 crew	(41)
1832	*America*	Sail	Convict transport	Unknown (likely United Kingdom to Australia)	180 days	175 crew and convicts	(42)
1918	HMAT *Boonah*	Steam	Troop ship	Durban, South Africa to Fremantle, Australia	17 days	164 crew, 931 troops	(43)

Table S4. Known outbreaks of influenza, smallpox, and measles 1850–1918

Year	Vessel	Type	Pathogen	Journey	Journey Duration	N	Outbreak Duration	Cases	Reference
1851	*Gold Hunter*	Steam	Smallpox	Panama – San Francisco	29 days	163	29+ days	1 case	(44)
1852	*Sir Charles Napier*	Sail	“Measles, dysentery, and fever”	Panama – San Francisco	90 days	211	“about three weeks” (newspaper article); 54 days (final death)	36 deaths (total for all outbreaks; no cause of death data available)	(45)

References

1. E Vynnycky, A Trindall, P Mangtani, Estimates of the reproduction numbers of Spanish influenza using morbidity data. *Int. J. Epidemiol.* **36**, 881–889 (2007).
2. LF White, M Pagano, Transmissibility of the Influenza Virus in the 1918 Pandemic. *PLOS ONE* **3**, e1498 (2008).
3. Anonymous, Another Instance of Pestilence Engendered in a Ship Crowded with Passengers from Ireland. *The Med. Repos.* **V**, 234–236 (1802).
4. F Marryat, *Mountains and Molehills, Or, Recollections of a Burnt Journal.* (Brown, Green, and Longman, London), (1855).
5. EJ Smith, 'Cleanse or Die': British Naval Hygiene in the Age of Steam, 1840–1900. *Med. Hist.* **62**, 177–198 (2018).
6. PE Sampson, "The lungs of a ship": Ventilation, acclimatization, and labor in the maritime environment, 1740–1800. *Hist. Sci.* **61** (2021).
7. T Wells, *The scale of medicines with which merchant vessels are to be furnished.* (John Churchill), (1861).
8. AL Gihon, Naval Hygeine in *A Reference Handbook of the Medical Sciences: Embracing the Entire Range of Scientific and Practical Medicine and Allied Science.* (William Wood & Company, New York), pp. 766–773 (1886).
9. IW Brewer, Report of Epidemic of 'Spanish Influenza', Which Occured at Camp A. A. Humphreys, VA., During September and October, 1918. *J. Lab. Clin. Medicine* **4**, 87–111 (1918).
10. SF Dudley, The Biology of Epidemic Influenza, Illustrated by Naval Experience. *Proc. Royal Soc. Medicine* **14**, 37–50 (1921).
11. J Cumpston, *The History of Small-pox in Australia, 1788-1908.* (Albert J. Mullett, Government Printer, Melbourne), (1914).
12. A Minooee, LS Rickman, Infectious Diseases on Cruise Ships. *Clin. Infect. Dis.* **29**, 737–743 (1999).
13. JM Miller, et al., Cruise Ships: High-Risk Passengers and the Global Spread of New Influenza Viruses. *Clin. Infect. Dis.* **31**, 433–438 (2000).
14. KS Willebrand, L Pischel, AA Malik, SM Jenness, SB Omer, A review of COVID-19 transmission dynamics and clinical outcomes on cruise ships worldwide, January to October 2020. *Eurosurveillance* **27** (2022).
15. JJ Regan, JS Vega, CM Brown, Infectious illnesses on cruise and cargo ships in *Infectious Diseases*, eds. E Petersen, LH Chen, P Schlagenhauf-Lawlor. (Wiley), 1 edition, pp. 35–44 (2017).
16. ER Cross, et al., Upper Respiratory Disease in Deployed U.S. Navy Shipboard Personnel. *Mil. Medicine* **157**, 649–651 (1992).
17. DM Vera, et al., Assessing the impact of public health interventions on the transmission of pandemic H1N1 influenza a virus aboard a Peruvian navy ship. *Influ. Other Respir. Viruses* **8**, 353–359 (2014).
18. KC Earhart, et al., Outbreak of influenza in highly vaccinated crew of U.S. Navy ship. *Emerg. Infect. Dis.* **7**, 463–465 (2001).
19. P Azimi, Z Keshavarz, JG Cedeno Laurent, B Stephens, JG Allen, Mechanistic transmission modeling of COVID-19 on the Diamond Princess cruise ship demonstrates the importance of aerosol transmission. *Proc. Natl. Acad. Sci.* **118**, e2015482118 (2021).
20. S Hatzianastasiou, et al., COVID-19 Outbreak on a Passenger Ship and Assessment of Response Measures, Greece, 2020. *Emerg. Infect. Dis.* **27**, 1927–1930 (2021).
21. H Maeda, et al., Epidemiology of Coronavirus Disease Outbreak among Crewmembers on Cruise Ship, Nagasaki City, Japan, April 2020. *Emerg. Infect. Dis.* **27** (2021).
22. T Veenstra, PD van Schelven, YM ten Have, CM Swaan, WMR van den Akker, Extensive Spread of SARS-CoV-2 Delta Variant among Vaccinated Persons during 7-Day River Cruise, the Netherlands. *Emerg. Infect. Dis.* **29**, 734–741 (2023).
23. MR Kasper, et al., An Outbreak of Covid-19 on an Aircraft Carrier. *New Engl. J. Medicine* **383**, 2417–2426 (2020).
24. PJ Hurtado, AS Kirosingh, Generalizations of the 'Linear Chain Trick': incorporating more flexible dwell time distributions into mean field ODE models. *J. Math. Biol.* **79**, 1831–1883 (2019).
25. HJ Wearing, P Rohani, MJ Keeling, Appropriate Models for the Management of Infectious Diseases. *PLOS Medicine* **2**, e174 (2005).
26. M Pineda-Krch, GillespieSSA: Implementing the Gillespie Stochastic Simulation Algorithm in R. *J. Stat. Softw.* **25**, 1–18 (2008).
27. J Lessler, et al., Incubation periods of acute respiratory viral infections: a systematic review. *The Lancet Infect. Dis.* **9**, 291–300 (2009).
28. LLH Lau, et al., Viral Shedding and Clinical Illness in Naturally Acquired Influenza Virus Infections. *The J. Infect. Dis.* **201**, 1509–1516 (2010).
29. M Biggerstaff, S Cauchemez, C Reed, M Gambhir, L Finelli, Estimates of the reproduction number for seasonal, pandemic, and zoonotic influenza: a systematic review of the literature. *BMC Infect. Dis.* **14**, 480 (2014).
30. FM Guerra, et al., The basic reproduction number (R 0) of measles: a systematic review. *The Lancet Infect. Dis.* **17**, e420–e428 (2017).
31. P Eicher, *Raising the Flag: America's First Envoys in Faraway Lands.* (University of Nebraska Press, Lincoln), (2018).
32. JE Stockdale, T Kypraios, PD O'Neill, Modelling and Bayesian analysis of the Abakaliki smallpox data. *Epidemics* **19**, 13–23 (2017).
33. V Costantino, MP Kunasekaran, AA Chughtai, CR MacIntyre, How Valid Are Assumptions About Re-emerging Smallpox?

A Systematic Review of Parameters Used in Smallpox Mathematical Models. *Mil. Medicine* **183**, e200–e207 (2018).
34. LJ Rasmussen, *San Francisco Ship Passenger Lists.* (San Francisco historic record & genealogy bulletin, Colma, CA) Vol. 2, (1965).
35. ME Willing, San Francisco Correspondence. *Sacramento Transcr.* (10 August, 1850).
36. A McGuinness, *Path of Empire: Panama and the California Gold Rush.* (Cornell University Press), (2007).
37. RH Fuson, *The Log of Christopher Columbus.* (McGraw-Hill Companies, New York), (1987).
38. ET Jones, MM Condon, J Cabot, *Cabot and Bristol's Age of Discovery: The Bristol Discovery Voyages 1480-1508.* (University of Bristol Press, Bristol, United Kingdom), (2016).
39. K Doherty, *Sea Venture: Shipwreck, Survival, and the Salvation of Jamestown.* (St. Martin's Publishing Group, New York), (2013).
40. R Fraser, *The Mayflower: The Families, the Voyage, and the Founding of America.* (St. Martin's Publishing Group, New York), (2017).
41. SlaveVoyages, Transatlantic Slave Trade Database: Voyage 92593, Diana, 1778 (2023).
42. BJ Paterson, MD Kirk, AS Cameron, C D'Este, DN Durrheim, Historical data and modern methods reveal insights in measles epidemiology: a retrospective closed cohort study. *BMJ Open* **3**, e002033 (2013).
43. JHL Cumpston, *Influenza and Maritime Quarantine in Australia.* (Issued under the Authority of the Minister for Trade and Customs, Albert J. Mullett, Government Printer, Melbourne), (1919).
44. Small Pox. *The Dly. Alta California* p. 2 (1851) 13 June.
45. Arrival of the Sir Charles Napier. – Dreadful Mortality. *The Alta California* p. 2 (1852) 22 May.
